# Stacked graph attention network hypertuned by Heteroscedastic and Evolutionary Bayesian Optimization for fault identification on nuclear power plants robust to sensor drift

**DOI:** 10.1038/s41598-026-60045-4

**Published:** 2026-07-03

**Authors:** Stefano Frizzo Stefenon, Kin-Choong Yow

**Affiliations:** Faculty of Engineering and Applied Sciences, University of Regina, Saskatchewan, S4S 0A2 Canada

**Keywords:** Bayesian optimization, Fault identification, Graph neural networks, Nuclear power plants, Engineering, Mathematics and computing

## Abstract

Fault identification in Nuclear Power Plants (NPPs) is critical for ensuring operational safety, reliability, and efficiency. Traditional diagnostic methods often rely on physical models and expert systems, which may struggle to capture the complex dynamics of transient events. To overcome these limitations, this paper proposes an optimized stacked Graph Attention Network (GAT) for fault detection in NPPs by modeling the complex interdependencies among system components as graphs. Transient operational data are transformed into graph representations, where nodes correspond to system variables, and edges capture physical relationships. The architecture of the proposed model is optimized using a Heteroscedastic and Evolutionary Bayesian Optimization (HEPO), ensuring the use of the best configuration. The proposed GAT-based model, hypertuned by HEPO, is trained to recognize patterns associated with both normal and faulty transient conditions, including sensor anomalies and actuator failures. Based on synthetic data generated from the Personal Computer Transient Analyzer (PCTRAN), the proposed model achieved results above 0.96 for accuracy, precision, recall, and F1-score in a statistical analysis.

## Introduction

The growing interest in the use of Nuclear Power Plants (NPPs), especially small modular reactors, requires robust fault identification mechanisms to ensure safety and operational reliability^1^. Transient analysis of reactor dynamics can be harnessed for the timely detection of anomalies and faults, particularly using malfunction simulators, thus avoiding critical conditions that could result in catastrophic failures^2^.

Fault identification is a critical component of safety management in NPPs, aiming to detect and diagnose malfunctions before they compromise reactor integrity and safety^3^. Traditional fault diagnosis techniques in NPPs often rely on model-based or statistical approaches that require expert knowledge, resulting in limited adaptability across varying operational scenarios. The high dimensionality and non-linear behavior of reactor systems further challenge these methods, motivating the exploration of data-driven Machine Learning (ML) approaches that can automatically extract relevant features from transient response data^4^.

Accident diagnosis and anomaly detection are related but distinct tasks in NPP monitoring. Accident diagnosis (fault diagnosis) focuses on classifying predefined fault conditions using labeled data, whereas anomaly detection aims to identify deviations from normal operation^5^. In practice, anomaly detection is used for early warning, while fault diagnosis provides precise fault categorization. In this work, the proposed model is formulated as a supervised fault identification (diagnosis) approach, while also being evaluated under anomaly-like conditions such as sensor faults^6^ and false positives^7^.

Reactor transients, characterized by time-dependent changes in key parameters such as neutron flux, coolant temperature, and pressure, provide information for early fault detection^8^. Data-driven transient analysis models have been developed to classify and predict transient events, with ML algorithms demonstrating high accuracy^9^. Deep Learning (DL) models are also applied in this regard, achieving promising fault identification rates^10^.

Transfer learning strategies have further enhanced model performance by leveraging pretrained DL models’ feature extractors and adapting them to NPP datasets, improving robustness across different fault types and operating conditions^11^. Despite these advances, challenges persist in ensuring model interpretability, handling noisy or incomplete data, and generalizing across multiple plant designs, highlighting the need for architectures that integrate domain knowledge with DL capabilities^12^.

Hybrid models currently stand out in applications involving DL due to their ability to combine features from different techniques in the same structure^13^. Data clustering techniques can aid fault diagnosis, improving the ability to make decisions in the event of a failure^14^. Besides the use of the state-of-the-art DL models for fault classification, the structure optimization can enhance the models’ performance, using methods such as Bayesian optimization^15^.

Despite the significant progress achieved by data-driven and deep learning approaches for fault identification in nuclear power plants, several limitations remain. Existing methods often struggle to accurately distinguish between true system faults and sensor-induced anomalies, particularly under transient and noisy conditions. In addition, many approaches fail to fully capture the complex interdependencies among system variables, which are critical for reliable fault diagnosis in safety-critical environments. These challenges limit the robustness and generalization capability of current models, especially when operating under uncertain or partially corrupted data.

To address these limitations, we propose a stacked Graph Attention Network (GAT) hypertuned by a Heteroscedastic and Evolutionary Bayesian Optimization (HEBO). The proposed model is evaluated using simulated datasets from the Personal Computer Transient Analyzer (PCTRAN), demonstrating improved performance in fault identification accuracy and early detection capabilities. Given that, the contributions of this paper are:

- Transient scenarios and fault conditions are simulated using the PCTRAN tool, and the study focuses on critical events such as loss-of-coolant accident, which is a major issue in NPPs.
- The paper proposes a deep learning model based on a stacked GAT that captures the complex interdependencies in NPP systems using graph representations of operational variables and their relationships. It applies HEBO to automatically configure the model architecture and hyperparameters, improving performance and generalization.
- The proposed model demonstrates improved performance compared to baseline methods under controlled simulation scenarios, indicating its potential effectiveness for fault identification tasks.
- Stability and robustness are demonstrated through 50 independent trials, given accuracy, precision, recall, and F1-score, showing minimal variation and all averaging above 0.96.

The proposed architecture models system variables as graph-structured data and leverages attention mechanisms to capture relevant relationships among them. The proposed method incorporates a data-driven graph construction strategy and a hypertuning framework to improve model adaptability and performance, enabling more accurate and robust fault identification in NPP systems.

The remainder of this paper is organized as follows: section “Related works” presents work related to fault identification in NPPs using ML and DL-based models. Section “Dataset” explains how the data set was generated to conduct the analysis in relation to the synthetic data. Section “Proposed methodology” presents the methodology proposed in this work. In section “Analysis and results”, the results are presented and discussed, and finally, section “Final remarks” presents final considerations.

## Related works

This session presents work related to fault identification in NPPs, considering the use of DL-based methods and graph models. DL^16^ and graph-based models are currently being widely used for fault classification because they automatically learn complex patterns in data and capture relationships between system components, making fault detection more accurate and robust^17^.

### DL-based models for NPP fault diagnosis

Fault and malfunction identification methods for NPPs can be grouped into model-based analytical approaches, signal-based techniques, data-driven Artificial Intelligence (AI) methods^5^, and hybrid strategies that combine physical models with DL. Model-based methods employ detailed reactor and system simulations to generate data for fault diagnosis, offering reliability since they are based on possible real faults^18^.

Signal-based techniques, such as wavelet, time-frequency, and spectral analysis, operate directly on measured vibration, temperature, pressure, flow, or acoustic signals to detect deviations from normal operation. Data-driven approaches leverage neural networks, support vector machines, decision trees, and deep architectures to learn fault signatures from historical data, achieving strong classification performance but often requiring large labeled datasets^19^. Hybrid and advanced methods, including Bayesian neural networks^20^, Convolutional Neural Network (CNN) with Long Short-Term Memory (LSTM) models^21^, deep transfer learning^22^, and even large language model-based explainability layers^23^, seek to balance accuracy, adaptability, and interoperability.

Domain discrepancy between training and testing conditions poses a significant challenge for the generalization of data-driven models in NPP fault diagnosis. CNNs trained on a single power level suffer from significant overfitting and limited ability to generalize across domains. Given this challenge, Lin et al.^24^ introduced an artificial disturbance method, which augments the training set with noise-disturbed data. This approach proves effective even with limited source domain diversity, showcasing its practical applicability in real-world fault diagnosis scenarios where data availability is often constrained.

The use of Generative Adversarial Networks (GANs) is a promising alternative for dealing with unbalanced data, which is a common problem in the analysis of faults in NPPs^25^, as applied by Gao et al.^26^ for sensor fault diagnosis, by Wang et al.^27^ for rotating machinery, and by Li et al.^28^ specifically for bearing-rotor systems.

Guo et al.^29^ introduced a convolutional variational autoencoder considering a GAN with a Random Forest (RF) to enhance fault diagnosis in NPPs. The approach begins with RF-based feature selection to filter out redundant measurements, followed by a variational autoencoder integrated with a GAN to generate fault samples given the imbalanced dataset. CNNs are then employed to extract deep features for intelligent diagnosis. Experimental validation using the Fuqing Unit 2 simulator demonstrates that their method significantly alleviates the effects of imbalanced data, suggesting its potential to advance reliable and efficient fault diagnosis systems in NPPs.

To build a robust fault detection framework, Zheng et al.^30^ applied the You Only Look Once (version 8 nano), refined by integrating efficient multi-scale attention blocks, FasterNet components, a slim-neck architecture, and DyHemail modules. These enhancements resulted in significant improvements in detection, achieving a mean average precision of 57.6% at Intersection over Union (IoU) 0.5 to 0.95 and 86.8% at IoU 0.5, all while preserving efficiency suitable for deployment on edge devices. The use of infrared images stood out as a solution to highlight system failures in thermal insulation glass fiber.

Li et al.^31^ propose a transfer learning approach that integrates Maximum Mean Discrepancy (MMD) with CNNs to minimize domain discrepancy in feature representation. By embedding the MMD-based distribution loss into the CNN’s objective function, the method enables more effective knowledge transfer from labeled source domains to unlabeled target domains under varying operating conditions. Experiments conducted using high-dimensional and nonlinear data demonstrate improvements in diagnostics across multiple tasks. These results suggest that their method has strong potential for enhancing the adaptability and reliability of fault diagnosis systems in NPP operations.

Fang et al.^32^ introduced an Enhanced Temporal Convolutional Network (ETCN) with self-attention and residual connections, whose hyperparameters are optimized by the Sparrow Search Algorithm (SSA). Tested on CPR1000 simulation data, with an F1-score equal to 0.9844, the proposed ETCN-SSA framework outperforms baseline temporal convolutional network, Gated Recurrent Unit (GRU), and LSTM models, demonstrating superior feature extraction and adaptive tuning for real-time diagnosis.

Li et al.^33^ combined Principal Component Analysis (PCA) for dimensionality reduction, time-series feature extraction to capture temporal trends, and convolutional neural networks for final classification. On a benchmark Pressurized Water Reactor (PWR) control-system dataset, the hybrid PCA-CNN scheme reduces detection latency and increases overall accuracy up to 99.8%. Wang et al.^34^ proposed a Convolutional Gated Recurrent Unit (CGRU) network that captures both spatial and temporal dependencies in multi-sensor streams, while an enhanced Particle Swarm Optimizer (PSO) tunes network hyperparameters for global optimality. Evaluated on simulated NPP transient data, the CGRU-PSO model achieves an average accuracy of 93.99% for 20 time steps.

Ayodeji et al.^35^ focused on enhancing the operator support system for the Chinese Qinshan II NPP. They applied PCA alongside two types of recurrent neural networks, Elman and Radial Basis Function (RBF), for fault detection and diagnosis. PCA was employed in the pre-diagnostic phase to filter noise from the data, addressing the complex and overlapping signal patterns typical of nuclear systems. The networks were trained and tested using fault scenario data from a PWR simulator. Results showed that the RBF network provided faster and more accurate fault diagnosis than the Elman network.

### Graph-based models for NPP fault diagnosis

Wang et al.^36^ proposed a method that combines spatiotemporal Graph Convolutional Networks (GCNs) with an autoencoder to extract and reconstruct the spatiotemporal information of system parameters. Anomalies are then detected based on trends in the reconstruction errors of the system’s operational data. Validation on two nuclear power system datasets demonstrates that this approach outperforms existing state-of-the-art methods, achieving a 5% higher accuracy in fault diagnosis and a 7.5% improvement in fault localization, while also providing earlier alerts by approximately three time steps.

Liu et al.^37^ introduced a hybrid model for multi-sensor fault detection in NPPs, combining GAT, adversarial autoencoder, and LSTM. The GAT captures sensor correlations, the autoencoder reconstructs data for fault indication, and the LSTM predicts expected sensor values. An fault score, derived from reconstruction and prediction errors, enables fault detection. Tests with simulated NPP data show improved accuracy and timeliness over existing approaches.

Zhang et al.^38^ proposed a Variational Graph Auto-Encoder (VGAE)-based fault diagnosis method for large-scale, unlabeled, and multi-source NPP data. Correlations are quantified via detrended cross-correlation analysis to build multivariate coupling networks, while symbolized difference sequences encode dynamic fluctuations. A semi-supervised VGAE then uses reconstruction loss as the fault indicator. Applied to an actual NPP circulating water system, the method outperforms traditional metrics by enhancing sensitivity and enabling earlier, more reliable fault diagnosis.

In^39^, the NPP data were labeled according to operating conditions and then used as inputs for a Graph Convolutional Network (GCN) for fault detection through graph classification. The authors analyze the sensitivity of the model to different parameters and configurations, highlighting how optimized configurations can improve performance. Experimental results demonstrate that the method achieves superior accuracy in fault identification, demonstrating both the feasibility and advantages of applying graph-based learning to safety management in NPPs.

Guohua et al.^40^ present a hybrid framework for fault detection in NPPs that combines threshold monitoring, Qualitative Trend Analysis (QTA), and Signed Directed Graph (SDG) inference. A multi-level threshold system generates reliable alarms, while SDG backward and forward inference identify and validate fault candidates. QTA further refines the diagnosis by assessing parameter trends. Simulations of critical accident scenarios show that this method enables faster and more accurate fault detection than conventional SDG-based approaches.

Zhang et al.^41^ presented a multi-relational graph-based framework for fault diagnosis of circulating water pumps in NPPs using multi-source data. To overcome limited fault samples, overlooked temporal dependencies, and sensor-related disruptions, the method integrates data generation, multi-period, and multi-mode blocks. These components augment samples, capture dependencies across time scales, and extract cross-sensor coupling, respectively. The fused representations are modeled with a multi-relational graph to capture rich spatial–temporal relationships. Experiments using their method demonstrate improvements in diagnostic accuracy over existing approaches.

Overall, the reviewed studies highlight graph-based models as a promising direction for fault detection and diagnosis in NPPs. By modeling correlations, dependencies, and couplings across sensors and data sources, methods such as GCNs, GATs, and VGAEs effectively capture complex spatial–temporal patterns that traditional time-series or statistical techniques often miss. These approaches improve the accuracy, robustness, and timeliness of fault diagnosis, even under conditions of multi-sensor faults, scarce samples, and nonlinear system dynamics. This indicates that graph-based learning offers a powerful framework to enhance safety management and operational reliability in NPPs.

## Dataset

This section introduces the PCTRAN, a NPP simulator used for training, education, and engineering analysis. It models a generic two-loop PWR, allowing real-time or accelerated simulations of normal operation, transients, and severe accidents. With customizable conditions and predefined malfunctions, PCTRAN provides a controlled environment for studying reactor behavior, making it a key tool for research and fault identification in NPPs^42^.

In this paper, false positives are modeled as faulty sensor readings inserted into normal operation to test whether sensor errors can be distinguished from real system faults. For data availability, all malfunction scenarios and baseline normal conditions were simulated and published in a public repository to ensure reproducibility and support future NPP fault detection studies. The term false positive refers to a situation where faulty sensor readings mimic fault conditions, potentially leading to incorrect fault identification^7^.

The proposed false positive construction is designed to approximate scenarios where sensor readings become inconsistent with the true system state. Specifically, measurements obtained under malfunction conditions are injected into normal operating data, creating inconsistencies between correlated variables. This setup does not aim to perfectly model all physical sensor failure mechanisms (e.g., gradual drift or calibration bias), but rather to simulate feature-level inconsistencies and cross-variable incoherence, which are common manifestations of sensor faults in practice.

In real NPP environments, sensor anomalies often lead to measurements that do not align with the physical relationships among system variables^43^. For example, a drifting or biased sensor may report values inconsistent with other correlated measurements, potentially triggering false alarms^44^. The proposed approach mimics this behavior by introducing signals that violate the learned relationships among variables, allowing the model to learn to rely on global system consistency rather than isolated measurements.

### Personal computer transient analyzer

The PCTRAN is a reactor transient simulation tool developed by Micro-Simulation Technology and adopted for NPP training, education, and engineering analysis. The PCTRAN provides an interactive graphical representation of the nuclear steam supply system, allowing users to model reactors under normal, transient, accident, and severe-accident conditions in real time or faster than real time^45^. Distributed by the International Atomic Energy Agency (IAEA) for a generic two-loop PWR, PCTRAN’s modular architecture supports detailed component-level malfunctions, customizable initial conditions, and flexible transient plotting, making it a staple in IAEA training workshops and research studies that compare its predictions against system codes^42^.

A PWR is a light?water nuclear reactor in which the primary coolant, ordinary $\hbox {H}_2$O, is maintained at a pressure high enough to keep it liquid at operating temperatures. Water in the reactor core removes heat from the fuel assemblies and moderates (slows) neutrons, facilitating sustained fission. The hot primary coolant then flows through steam generators, transferring heat to a secondary loop where steam is produced under lower pressure, driving turbines to generate electricity^46^.

**Fig. 1:**
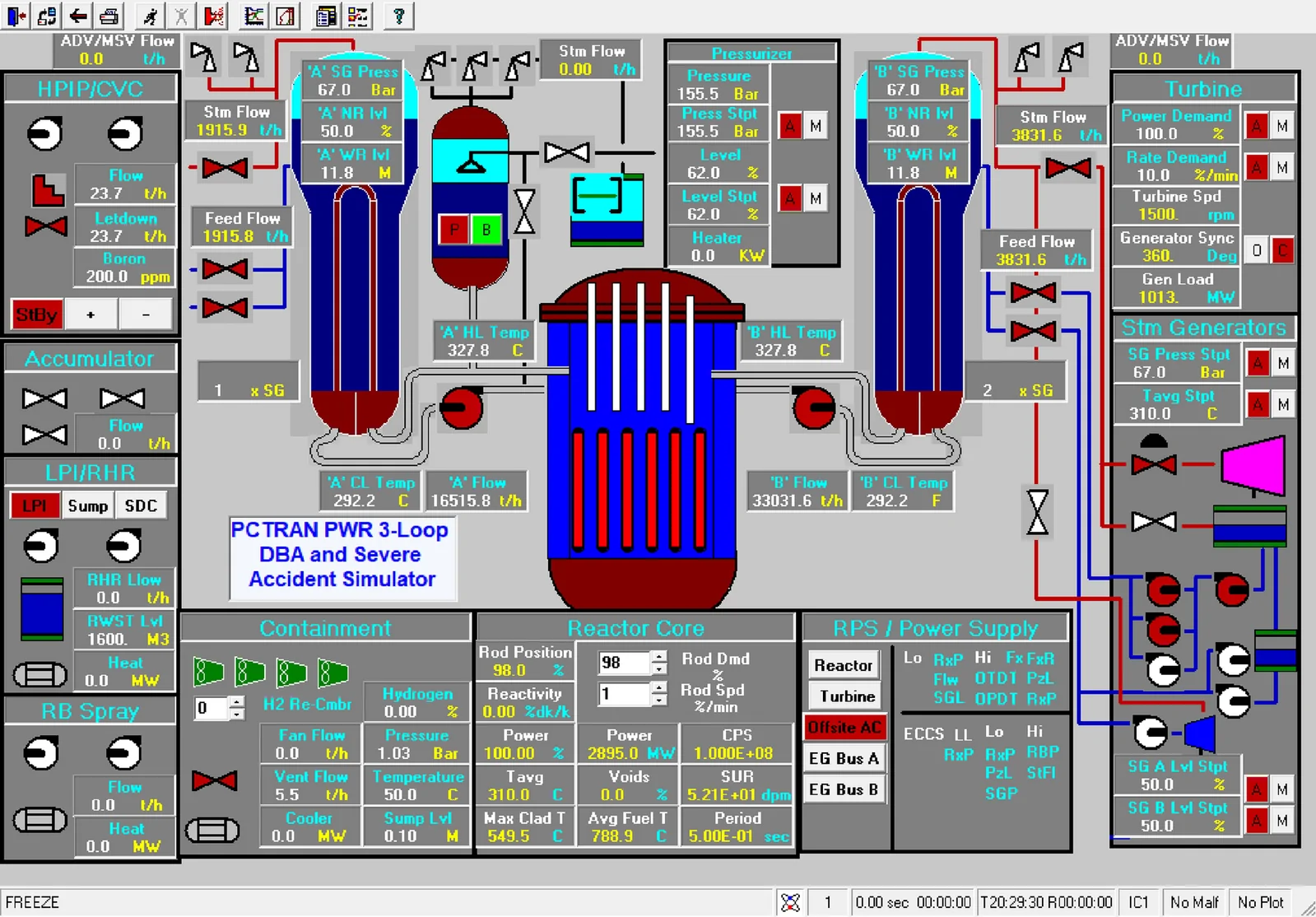
PCTRAN simulator interface.

Figure 1 presents the PWR system in the PCTRAN simulator version 7.0.1. In the PCTRAN, users can run scenarios at real-time or up to 16*×* faster to quickly explore long transients. Considering that the focus of this paper is to explore fault identification on NPP, the simulated malfunctions are the focus of the analysis. This simulator triggers up to 20 reactor-system malfunctions from a menu or component-specific faults via right-click menus, with visual indicators of active malfunctions^47^.

The possible simulated malfunctions are: (1) Loss of coolant accident (hot leg); (2) Loss of coolant accident (cold leg); (3) Steam line break inside containment; (4) Steam line break outside containment; (5) Spark presence for hydrogen burn; 6) Loss of Alternating Current (AC) power; (7) Loss of flow (locked rotor); (8) Anticipated transient without scram; (9) Turbine trip; (10) Steam generator: A tube rupture; (11) Steam generator: B tube rupture; (12) Rod withdrawal/insertion; (13) Reserved for future; (14) Moderator dilution; (15) Load rejection; (16) Containment failure or spark for $\hbox {H}_2$ detonation; (17) Fuel failure at power; (18) Fuel handling accident in containment; (19) Fuel handling accident in auxiliary building; (20) Letdown line break in auxiliary building^42^.

The most frequently encountered thermonuclear operational or accident-related issue among the ones listed typically varies by plant design and operating history^48^. However, based on data from nuclear safety studies, operating experience, and risk assessments, some events are significantly more frequent than others^49^.

### False positives

**Fig. 2:**
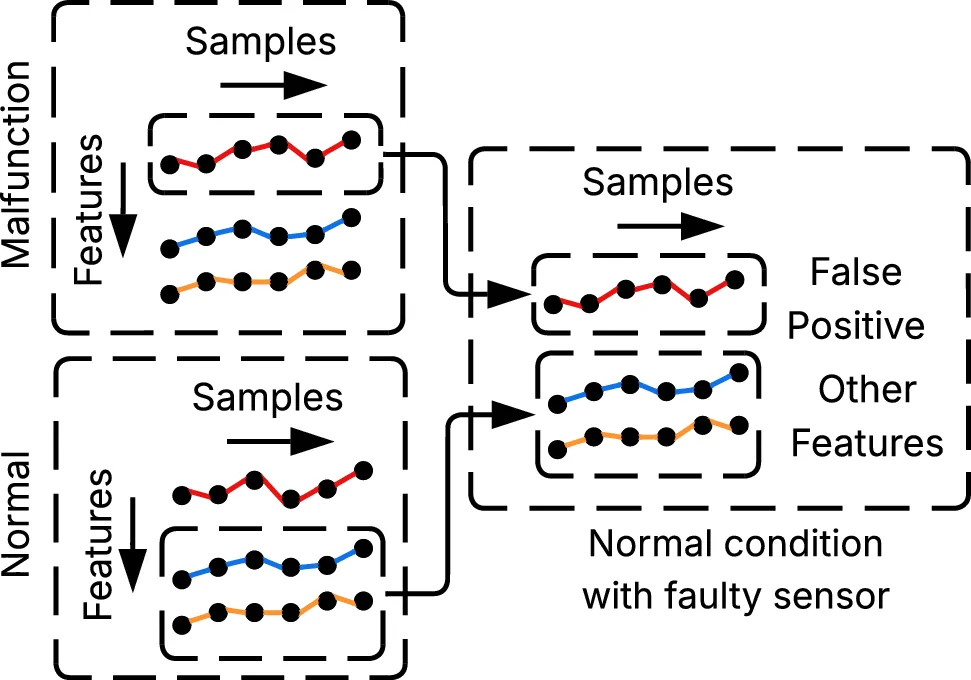
Strategy for simulating sensor-induced false positives.

Figure 2 presents how the false positives are created. On the left, the top section shows malfunction features, while the bottom shows normal features, both represented by time-series samples. In this work, we defined samples as the number of measurements recorded, considering the PCTRAN saved variables available in Appendix A.

During dataset creation, a subset of normal condition samples is intentionally combined with malfunction data representing faulty sensor outputs.

As a result, these synthetic samples represent normal operating conditions affected by sensor faults. Although some measurements exhibit fault-like behavior, the physical state of the NPP remains normal. These samples (called false positives) emulate situations in which a faulty sensor produces measurements similar to those observed during an accident. Such situations may lead a diagnostic system to incorrectly identify a plant fault when the plant is actually operating normally, resulting in a false alarm.

For the selected variables, measurements obtained from PCTRAN simulations under faulty sensor conditions are inserted into samples corresponding to normal plant operation. Consequently, the resulting synthetic samples contain abnormal sensor readings while the actual operating condition of the NPP remains normal. These samples emulate sensor-induced anomalies that may resemble accident conditions and potentially trigger false fault alarms in diagnostic systems. After creating synthetic samples, the data is loaded as a graph into the proposed model. The methodology of the proposed model is presented in the next section.

The generated synthetic sample represents a partially faulty condition, where only this feature exhibits fault characteristics while all other features remain normal. If the model classifies this sample as a fault, it suggests that the abnormal behavior of a single feature is sufficient to trigger an alarm, even though the overall operating condition is predominantly normal. This behavior mimics a false positive event in fault detection systems.

All analyses consider the comparison between normal operating conditions and normal operating conditions affected by sensor-induced anomalies. The objective is to correctly classify samples containing faulty sensor measurements as normal operating conditions whenever the underlying plant state is normal. This capability allows the diagnostic system to distinguish actual NPP faults from anomalies caused solely by sensor malfunction.

This would be used in an NPP to distinguish a normal condition in which the problem occurs specifically in a drifting sensor and not in the entire system.

The dataset was designed to emulate the identification of sensor faults to accident diagnosis in NPPs under realistic, real-time conditions. This design enables the evaluation of diagnostic performance under uncertainty and the analysis of mitigation strategies, as described in^50^.

### Data availability

To ensure the reproducibility of the experiments presented in this paper, all malfunction (failure) situations were simulated up to 10 minutes after their occurrence, considering the use of extreme parameters (1,000% of normal) to simulate critical conditions related to the problems that can occur at the power plant. These experiments are available at: https://github.com/SFStefenon/NPPmalfunctions.

To have a baseline condition for comparison, a simulation of the NPP under normal conditions was also carried out and is available to be used in future comparisons. Appendix A presents the variables saved in the experiments. Besides the experiments conducted to create this database, the algorithms proposed in this work (as discussed in the next section) are available to be compared for other tasks that can be applied to fault detection in NPP.

## Proposed methodology

In this section, the methodology proposed in this paper is explained in detail. Subsection “Data normalization” explains how the data is loaded and normalized. In subsection “Stacked graph attention network”, the base architecture of the stacked GAT is explained. This provides the foundation for creating a customized model using HEBO, which is explained in subsection “GAT layer”.

### Data normalization

During data loading, the path to the directory containing the dataset files is defined, along with the number of splits used for cross-validation. The features are standardized to improve model convergence and performance, while the target labels are encoded to ensure they are in a consistent numerical format.

Normalization is applied by centering the data (zero mean) and scaling it to unit variance, which enhances the effectiveness of training. The class labels are transformed into numerical form using label encoding, ensuring compatibility with algorithms that require categorical targets to be represented as integers. The final output consists of the standardized features and the encoded labels.

### Stacked graph attention network

We propose a stacked GAT architecture with a configurable depth, where the graph structure is constructed using a data-driven K-Nearest Neighbors (K-NN) strategy. Nodes correspond to individual samples, and edges encode similarity relationships in the feature space based on distances computed from the training set. The resulting graph remains fixed during training. Each GAT layer leverages attention mechanisms to adaptively weight neighboring nodes, enabling the model to emphasize the most informative relationships during feature propagation.

The number of layers is treated as a configurable parameter, allowing the model to be easily adapted for shallow or deep graph reasoning tasks. This flexible and data-driven architecture is particularly well-suited for applications where the graph structure is implicit or evolving, such as in spatiotemporal data analysis, which is the task covered here.

Graph Neural Networks generalize neural architectures to graph-structured data. Among them, the GAT introduces an attention mechanism to learn the importance of neighboring nodes during message aggregation^37^. In this paper, we present a stacked GAT that uses a variable number of layers and constructs its graph dynamically using K-NN, allowing flexible application in tasks where the input data is not naturally a graph.

Given a dataset ${\mathcal {X}} = \{ \textbf{x}_1, \dots , \textbf{x}_N \} \subset {\mathbb {R}}^F$, where each $\textbf{x}_i$ is a feature vector, we construct a directed graph ${\mathcal {G}} = ({\mathcal {V}}, {\mathcal {E}})$ using K-NN^51^. For each node *i*, define its neighborhood:

1 $$\begin{aligned} {\mathcal {N}}_i = \{ j \in {\mathcal {V}} \setminus \{i\} \mid \textbf{x}_j \text { is among the } K \text { nearest neighbors of } \textbf{x}_i \}. \end{aligned}$$

Edges are encoded via a sparse adjacency list as an edge_index tensor:

2 $$\begin{aligned} \texttt {edge\_index} = \begin{bmatrix} i_1 & i_2 & \dots \\ j_1 & j_2 & \dots \end{bmatrix}, \quad \text {where } (i_k, j_k) \in {\mathcal {E}}. \end{aligned}$$

#### GAT layer

Let $\textbf{h}_i^{(\ell )} \in {\mathbb {R}}^{F_\ell }$ be the representation of node *i* at layer *ℓ*. The GAT layer computes:

3 $$\begin{aligned} \textbf{z}_i^{(\ell )} = \textbf{W}^{(\ell )} \textbf{h}_i^{(\ell )} \in {\mathbb {R}}^{F_{\text {out}}}. \end{aligned}$$

The attention coefficient between node *i* and *j* is:

4 $$\begin{aligned} e_{ij}^{(\ell )} = \text {LeakyReLU} \left( \textbf{a}^{(\ell )\top } [\textbf{z}_i^{(\ell )} \, \Vert \, \textbf{z}_j^{(\ell )}] \right) \end{aligned}$$

where *‖* denotes vector concatenation, and $\textbf{a}^{(\ell )} \in {\mathbb {R}}^{2F_{\text {out}}}$ is a learnable parameter. These coefficients are normalized:

5 $$\begin{aligned} \alpha _{ij}^{(\ell )} = \frac{\exp (e_{ij}^{(\ell )})}{\sum _{k \in {\mathcal {N}}_i} \exp (e_{ik}^{(\ell )})}. \end{aligned}$$

In hidden layers, attention is applied in parallel across *H* layer, and their outputs are concatenated:

6 $$\begin{aligned} \textbf{h}_i^{(\ell +1)} = \mathbin \Vert _{h=1}^{H} \sigma \left( \sum _{j \in {\mathcal {N}}_i} \alpha _{ij}^{(\ell , h)} \textbf{W}^{(\ell , h)} \textbf{h}_j^{(\ell )} \right) , \quad \text {for } \ell < L - 1. \end{aligned}$$

Each layer has independent weights $\textbf{W}^{(\ell , h)} \in {\mathbb {R}}^{F_{\text {out}} \times F_\ell }$. The output dimension of layer *ℓ +1* becomes $H \cdot F_{\text {out}}$ due to concatenation^52^.

In the final layer *ℓ = L - 1*, only one layer is used and outputs are averaged:

7 $$\begin{aligned} \textbf{h}_i^{(L)} = \sigma \left( \sum _{j \in {\mathcal {N}}_i} \alpha _{ij}^{(L-1)} \textbf{W}^{(L-1)} \textbf{h}_j^{(L-1)} \right) . \end{aligned}$$

This final output is passed through a log_softmax activation:

8 $$\begin{aligned} \hat{\textbf{y}}_i = \log \left( \frac{\exp (\textbf{h}_i^{(L)}[c])}{\sum _{c'} \exp (\textbf{h}_i^{(L)}[c'])} \right) , \quad \forall c \in \{1, \dots , C\}. \end{aligned}$$

#### Training

The model is trained using the Adam optimizer, which adapts learning rates per parameter using first and second moments of the gradients. For each parameter $\theta _t$ at training step *t*:

9 $$\begin{aligned} \begin{aligned} m_t&= \beta _1 m_{t-1} + (1 - \beta _1) \nabla _{\theta } {\mathcal {L}}_t \\ v_t&= \beta _2 v_{t-1} + (1 - \beta _2) (\nabla _{\theta } {\mathcal {L}}_t)^2 \\ {\hat{m}}_t&= \frac{m_t}{1 - \beta _1^t}, \quad {\hat{v}}_t = \frac{v_t}{1 - \beta _2^t} \\ \theta _{t+1}&= \theta _t - \eta \frac{{\hat{m}}_t}{\sqrt{{\hat{v}}_t} + \epsilon } \end{aligned} \end{aligned}$$

where $\beta _1$, $\beta _2$, *ε*, *η* are hyperparameters of the optimizer^53^.

The model is trained using negative log-likelihood loss, given by:

10 $$\begin{aligned} {\mathcal {L}} = - \sum _{i \in {\mathcal {T}}} \log {\hat{y}}_{i, y_i} \end{aligned}$$

where ${\mathcal {T}}$ is the training mask, and $y_i$ is the true label.

During training, dropout with probability *p=0.5* is applied after each GAT layer (except the final one):

11 $$\begin{aligned} \textbf{h}_i^{(\ell +1)} = \text {Dropout}\left( \textbf{h}_i^{(\ell +1)} \right) , \quad \text {for } \ell < L - 1. \end{aligned}$$

#### Deeper architecture

Our architecture supports a dynamic number of stacked GAT layers (*L*) defined by HEBO (explained in the next subsection). The final representation is recursively defined as:

12 $$\begin{aligned} \textbf{H}^{(L)} = \text {GAT}^{(L)} \left( \dots \text {GAT}^{(2)} \left( \text {GAT}^{(1)}(\textbf{X}) \right) \dots \right) . \end{aligned}$$

**Fig. 3:**
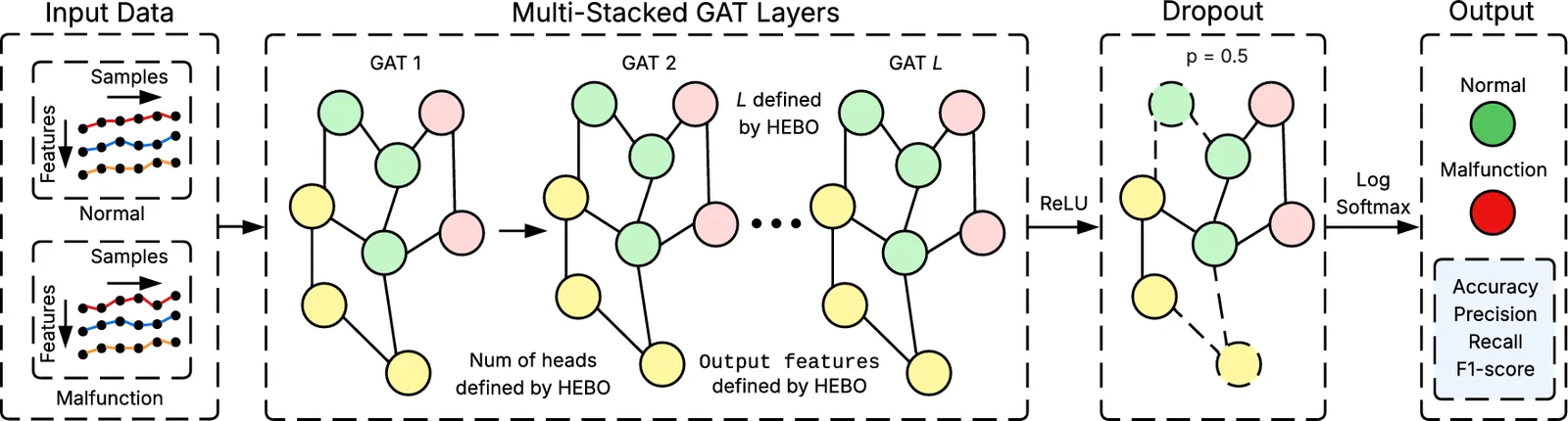
Proposed stacked GAT structure.

The variation of the number of layers allows for controlled depth depending on the dataset complexity and task requirements. Figure 3 illustrates a multi-staged pipeline for malfunction detection to explain our proposed structure. Input data consists of time-series features from both normal and malfunctioning samples. These features are structured as graph nodes and passed through a series of multi-stacked GAT layers, where each layer uses multiple attention heads to learn node representations.

The number of heads, output feature size, and total layers *L* are determined via HEBO (a hyperparameter optimization method). After the final GAT layer, node embeddings pass through a ReLU activation and a dropout layer to prevent overfitting. The resulting representations are then classified using a log-softmax function to predict either normal (green) or malfunction (red) states. Model performance is evaluated using metrics such as accuracy, precision, recall, and F1-score. The model performs binary classification, distinguishing between normal operation with all sensors functioning correctly and normal operation affected by a faulty sensor measurement.

### Heteroscedastic and Evolutionary Bayesian Optimization (HEBO)

Bayesian optimization addresses the problem of finding the global maximum of a black-box function $f: {\mathcal {X}} \rightarrow {\mathbb {R}}$ over a bounded domain ${\mathcal {X}} \subset {\mathbb {R}}^d$:

13 $$\begin{aligned} \text {arg max}_{x \in {\mathcal {X}}} f(x). \end{aligned}$$

where *x* is a configuration choice, ${\mathcal {X}}$ is a (potentially) mixed design space, and *f*(*x*) is a validation accuracy to maximize.

At each iteration *i*, the algorithm selects a batch of *q* points $x^{\text {new}}_{1:q} \in {\mathcal {X}}^q$ and observes ${y}^{\text {new}}_{1:q} = f(x^{\text {new}}_{1:q})$. The Bayesian optimization proceeds in two steps: (i) constructing a surrogate model, typically a Gaussian Process (GP), and (ii) maximising an acquisition function to propose new query points^54^.

The GP surrogate is defined by a mean function *m*(*x*) and covariance function $k_\theta (x, x')$, where *θ* are kernel hyperparameters. Observations are modelled as $y_\ell = f(x_\ell ) + \epsilon _\ell$ with $\epsilon _\ell \sim {\mathcal {N}}(0, \sigma ^2_{\text {noise}})$. The posterior predictive distribution over a batch of query points $x_{1:q}$ given dataset ${\mathcal {D}} = \{x, y\}$ is:

14 $$\begin{aligned} \begin{bmatrix} y \\ f(x_{1:q}) \end{bmatrix} \mid \theta \sim {\mathcal {N}} \begin{bmatrix} m(x) \\ m(x_{1:q}) \end{bmatrix}, \begin{pmatrix} K_\theta + \sigma ^2 I & k_\theta (x_{1:q}) \\ k_\theta ^\top (x_{1:q}) & k_\theta (x_{1:q}, x_{1:q}) \end{pmatrix} , \end{aligned}$$

from which the posterior mean and covariance are:

15 $$\begin{aligned} \mu _\theta (x_{1:q})&= m(x_{1:q}) + k_\theta (x_{1:q})^\top (K_\theta + \sigma ^2 I)^{-1}(y - m(x)), \end{aligned}$$

16 $$\begin{aligned} \Sigma _\theta (x_{1:q})&= k_\theta (x_{1:q}, x_{1:q}) - k_\theta (x_{1:q})^\top (K_\theta + \sigma ^2 I)^{-1} k_\theta (x_{1:q}). \end{aligned}$$

To balance exploration and exploitation, the following acquisition functions for the Expected Improvement (EI) are commonly used:

17 $$\begin{aligned} \alpha ^{\theta }_\text {EI}(x_{1:q} \mid {\mathcal {D}}) = {\mathbb {E}}_{\text {post.}} \left[ \max _{j \in 1:q} \left\{ \text {ReLU}(f(x_j) - f(x^+)) \right\} \right] , \end{aligned}$$

where $x^{+}$ is the best observed input. The Probability of Improvement (PI) is given by:

18 $$\begin{aligned} \alpha ^{\theta }_\text {PI}(x_{1:q} \mid {\mathcal {D}}) = {\mathbb {E}}_{\text {post.}} \left[ \max _{j \in 1:q} \left\{ 1\!\!1_{\{f(x_j) > f(x^+)\}} \right\} \right] , \end{aligned}$$

and the Upper Confidence Bound (UCB) is:

19 $$\begin{aligned} \alpha ^{\theta }_\text {UCB}(x_j) = {\mathbb {E}}_{\text {post.}} \left[ \max _{j \in 1:q}\left[ \mu _\theta (x_j) + \sqrt{\frac{\beta \pi }{2}} \mid \gamma _\theta (x_j) \mid \right] \right] . \end{aligned}$$

where $\mu _{{\theta }}$ is the posterior mean, and $\gamma _{\theta }(x_{j}) = f(x_{j}) - \mu _{\theta }(x_{j})$.

HEBO, proposed by Cowen-Rivers et al.^55^, combines multiple acquisition functions into a multi-objective optimization:

20 $$\begin{aligned} \max _{x} \left( \alpha ^{-\theta }_\text {EI}(x \mid {\mathcal {D}}), \alpha ^{-\theta }_\text {PI}(x \mid {\mathcal {D}}), \alpha ^{-\theta }_\text {UCB}(x \mid {\mathcal {D}}) \right) . \end{aligned}$$

where $\alpha ^{-\theta }_\text {type}(x \mid {\mathcal {D}})$ is the robust acquisition function for EI, PI, and UCB. This approach yields a Pareto-efficient set of candidate solutions across acquisition strategies.

When incorporating input/output transformations (e.g., Box-Cox see Eq. (23) or Yeo-Johnson according to Eq. (24)), to handle non-stationarity and heteroscedasticity, the GP hyperparameters *θ* and transformation parameters *γ* are learned via:

21 $$\begin{aligned} \max _{\theta , \gamma } -\frac{1}{2} T_{\zeta ^*}(y)^\top (K_\theta ^\gamma + \sigma _{\text {noise}} I)^{-1} T_{\zeta ^*}(y) - \frac{1}{2} \mid K_\theta ^\gamma + \sigma ^2_{\text {noise}}I\mid - \text {const}, \end{aligned}$$

where $T_{\zeta ^*}$ denotes the optimal output transformation, and $K_\theta ^\gamma$ is the kernel matrix under the Kumaraswamy-warped inputs.

The Kumaraswamy transformation per dimension *k* is given by:

22 $$\begin{aligned} {[}\text {Kumaraswamy}_{{\gamma }}(x_{l})]_{k} = 1 - (1 - [x_{l}]_{k}^{a_{k}})^{b_{k}}, \quad \forall k \in [1:d] \end{aligned}$$

where *γ* is the vector concatenating all parameters, such as $a_k$ and $b_k$ which are learnable parameters.

The Box-Cox transformation^56^ is given by:

23 $$\begin{aligned} \log \left[ \sum _{l=1}^{n} \frac{\left( \textrm{T}_{\zeta }(y_{l}) - \overline{\textrm{T}}_{\zeta }({y})\right) ^{2}}{n} \right] ^{\frac{n}{2}} + \sum _{l=1}^{n} \log \left[ \textrm{T}_{\zeta }(y_{l})\right] ^{(1-\zeta )} \end{aligned}$$

where $\textrm{T}_{\zeta }(y_{l})$ is sample mean of the transformed labels and *n* is the number of datapoints. Yeo-Johnson (YJ) transformation^57^ is given by:

24 $$\begin{aligned} \textrm{YJ}_{\zeta }(y_{l}) = {\left\{ \begin{array}{ll} \frac{(y_{l} + 1)^{\zeta } - 1}{\zeta }, & \text {if } \zeta \ne 0,\ y_{l} \ge 0 \\ \log (y_{l} + 1), & \text {if }\zeta = 0,\ y_{l} \ge 0 \\ \frac{(1 - y_{l})^{2-\zeta } - 1}{\zeta - 2}, & \text {if }\zeta \ne 2,\ y_{l}< 0 \\ -\log (1 - y_{l}), & \text {if }\zeta = 2,\ y_{l} < 0. \end{array}\right. } \end{aligned}$$

To evaluate the hypertuning setup, the performance over the trials is analyzed. A trial refers to a single execution of the model training and evaluation process using a specific combination of hyperparameters. Each trial tests one configuration drawn from the search space, allowing the optimizer to assess the model’s performance under those settings. The outcome of a trial is recorded through the model’s performance, which is then compared across all trials to identify the best-performing configuration^58^.

The search space is defined to guide the hyperparameter optimization process by specifying the range of values that key architectural components of the model can assume. In this work, the search space for output_features is from 2 to 64, for heads is from 2 to 16, and layers is from 2 to 16, enabling the evaluation of different model capacities and architectural depths within these predefined bounds.

## Analysis and results

This section will present the results of the analysis of models for classifying faults in NPPs. The main goal of this evaluation is to identify faults considering incorrect measurements, since NPP operation cannot be vulnerable to a sensor failure resulting in an incorrect measurement. Given this condition, the analysis is based on the evaluation of false positives (the measurement incorrectly indicates that there is a fault when there is not).

The dataset used in this section is composed of simulated malfunction scenarios generated using the PCTRAN NPP simulator. A total of up to 20 predefined fault types are available, including loss-of-coolant accidents, steam generator failures, turbine trips, and electrical faults. Each malfunction is simulated under extreme operating conditions, reaching up to 1000% of nominal values, and recorded for a duration of up to 10 minutes after fault initiation to capture transient system behavior. To simulate a condition in which there is limited data to analyze, five features are considered.

In this study, the analysis is structured in two parts. First, subsections 5.2 to 5.5 focus on a representative critical fault, namely the loss-of-coolant accident (hot leg), to evaluate the model behavior under controlled conditions. Second, subsection 5.6 evaluates the generalization capability of the model across multiple fault types, including loss of AC power, steam generator tube rupture, steam line break inside containment, and letdown line break in the auxiliary building.

The dataset includes synthetic sensor fault conditions (false positives), where measurements from faulty sensors are injected into normal operation data. This configuration enables the evaluation of the model under uncertainty, requiring it to distinguish between actual system faults and sensor-induced anomalies.

The loss-of-coolant accident in the hot leg of an NPP refers to a severe scenario in which a rupture or significant leakage occurs in the hot leg pipe that carries high-temperature coolant from the reactor vessel to the steam generators. Since the hot leg operates at high pressure and temperature, such a break can lead to a rapid decrease in reactor coolant inventory, impairing the core’s ability to remove heat^59^.

This situation poses a serious safety challenge because the exposed fuel rods may overheat if coolant circulation is not promptly restored. Emergency core cooling systems are therefore activated to inject borated water into the reactor vessel, compensating for the loss of coolant and maintaining core cooling. The safety analysis of the hot leg is a critical aspect of reactor design and licensing, as it ensures that even under these extreme conditions, the plant can be safely shut down without significant fuel damage or release of radioactive material^60^.

### Evaluation metrics and configuration

The algorithm is implemented in Python using PyTorch Geometric. All experiments are performed using Google Colab based on a NVIDIA T4 graphics processing unit with 16 GB of memory. Experiments were conducted for up to 100 epochs, considering k-fold cross-validation. For a comparative analysis, in this study, the accuracy, precision, recall, and F1-score metrics were considered, given by:

25 $$\begin{aligned} \text {Accuracy}&= \frac{TP + TN}{TP + TN + FP + FN} \end{aligned}$$

26 $$\begin{aligned} \text {Precision}&= \frac{TP}{TP + FP} \end{aligned}$$

27 $$\begin{aligned} \text {Recall}&= \frac{TP}{TP + FN} \end{aligned}$$

28 $$\begin{aligned} \text {F1-score}&= 2 \times \frac{\text {Precision} \times \text {Recall}}{\text {Precision} + \text {Recall}} \end{aligned}$$

where *TP* are the True Positives, *TN* are the True Negatives, *FP* are the False Positives, and *FN* are the False Negatives.

The evaluation metrics consider the identification of false positives, in which a synthetic experiment that includes false positives is classified correctly, regardless of the false positives that represent faulty sensors. The evaluation is conducted using synthetic data generated by the PCTRAN simulator, providing a controlled environment for preliminary validation of the proposed approach.

All methods included in the comparative analysis were evaluated under the same conditions, using the same set of features and the same type of input data. Cross-validation was performed consistently and equivalently across all methods compared to the proposed model. In this study, only the proposed method was fine-tuned using HEPO, since the other well-established methods were used as baseline models for a comparative analysis.

### Hypertuning analysis

To define the hyperparameters of the model proposed in this paper, we first conduct a comparative analysis with several types of surrogate models for HEBO implementation, namely: GP using GPy (GPY), default GP using BoTorch (GP), RF, GP with Matern kernel (GP Matern), GP with RBF kernel (GP RBF), linear regression model (linear), and Multi-task Gaussian Process (MTGP). In this evaluation, a k-fold equal to 5 splits is used, considering 50 samples and 10 variables.

**Fig. 4:**
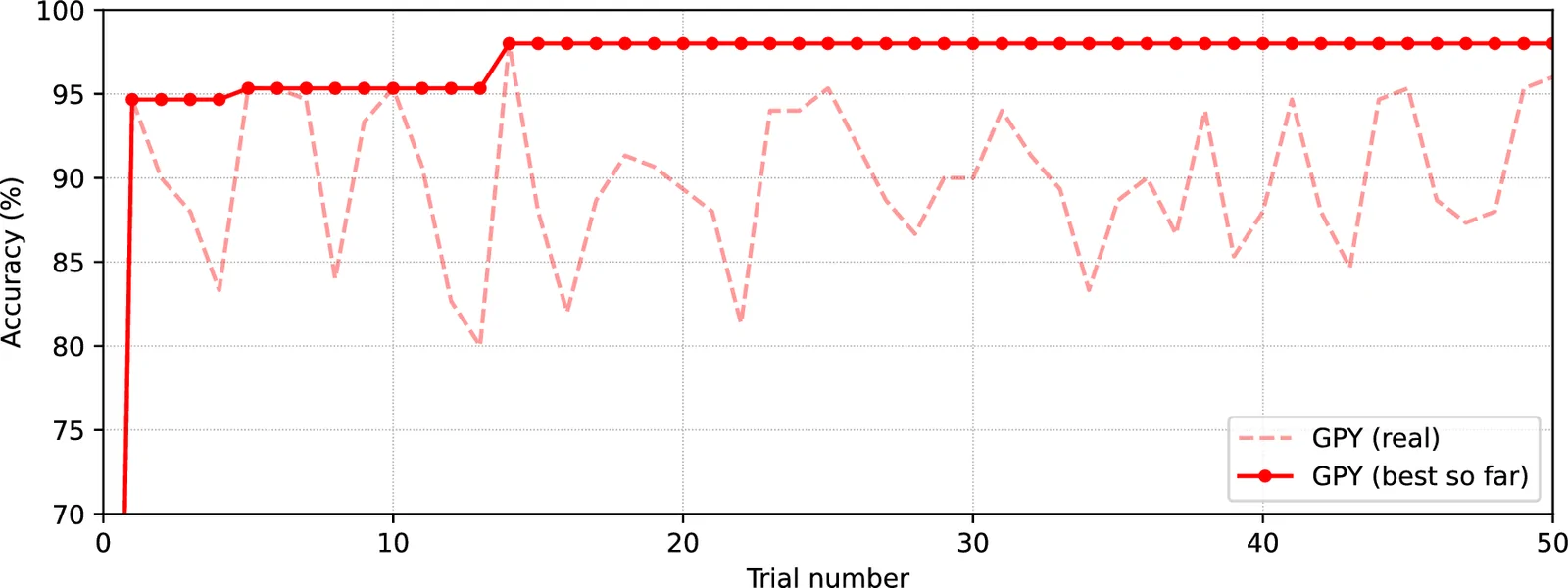
Accuracy of our method during hypertuning by HEBO: best so far versus observed (real) results per trial.

Figure 4 shows the comparison between the results of the model’s trials to find the best result and the best result compared to previous trials. During the optimization process, the model iteratively searches for a set of hyperparameters that, when combined, result in the model configuration. The best result of this configuration is saved for later use.

**Fig. 5:**
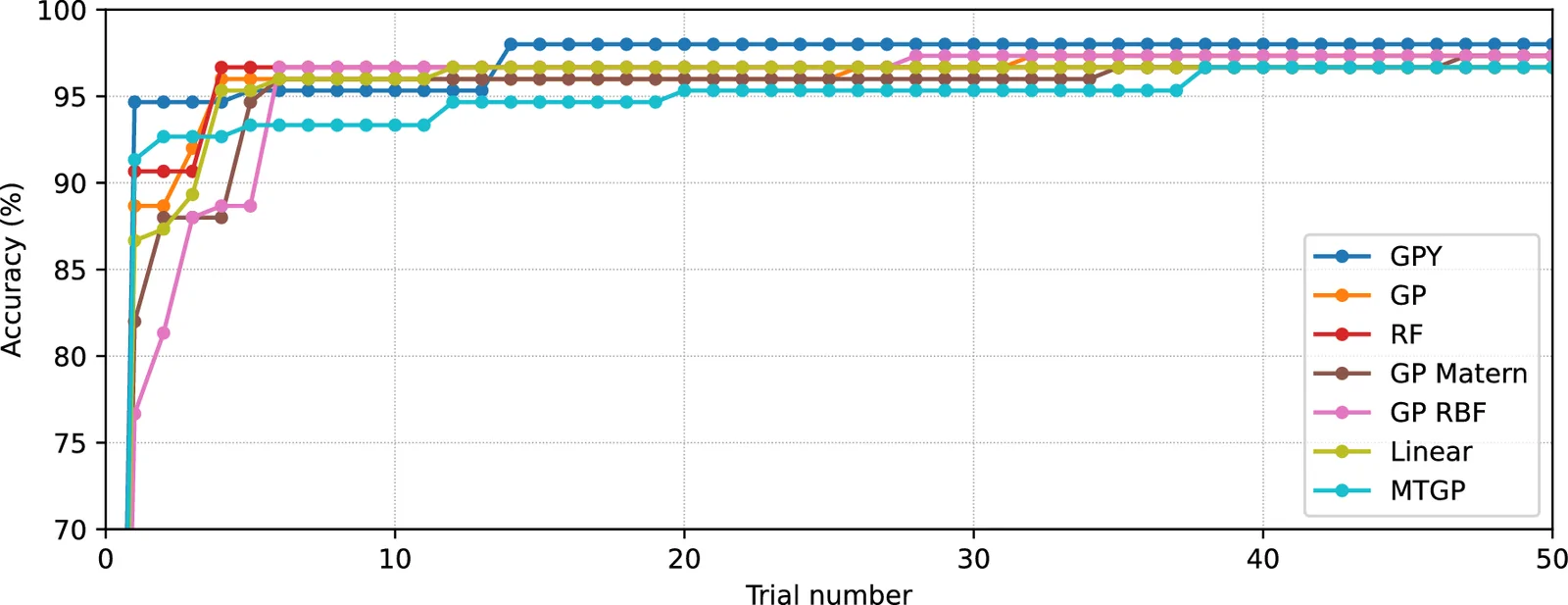
Accuracy of our method using different types of surrogate models for HEBO implementation (best results so far).

Figure 5 shows the best accuracy results for each trial, considering the evaluated types of surrogate models. The best accuracy is the one that had the highest values compared to previous trials. In this plot, most models achieved accuracy results better than 80% after the first trial. The best result (accuracy of 98.0%) was achieved using the GPY hypertuning strategy, which considered 5 heads with 58 output_features per attention layer, utilizing 8 layers. This hyperparameter configuration is used to have an optimized model.

This result shows that the GPY surrogate model was the best strategy for using HEBO, proving that the choice of strategy was appropriate for model hyperparameter tuning. In addition to its high performance, this strategy also proved to be the most efficient, as it achieved this performance in fewer attempts compared to other methods.

**Fig. 6:**
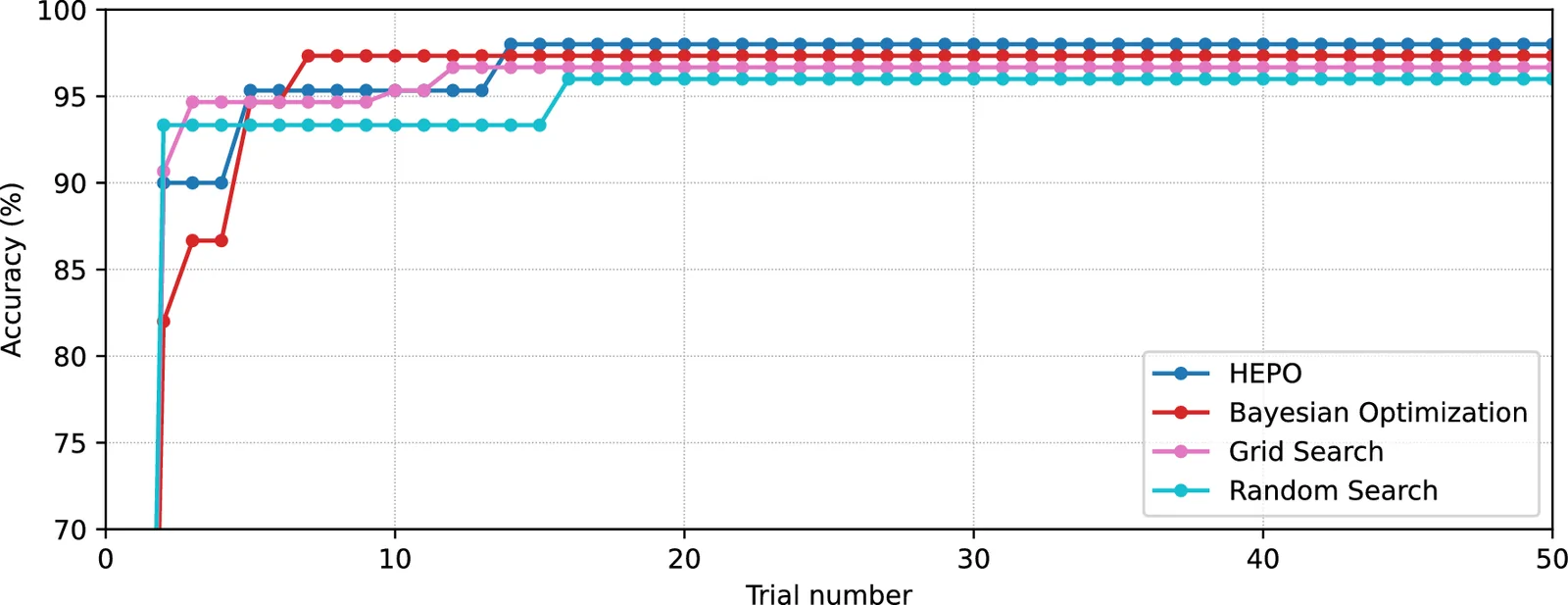
Accuracy of different hypertuning methods (best results so far).

To further evaluate the HEBO, we compare it to well-established hypertuning strategies, namely Bayesian optimization, grid search, and random search. Figure 6 presents the results of these evaluations considering 50 trials. HEPO exhibits the best results, reaching approximately 98% accuracy within the first few trials.

Bayesian Optimization presents a strong performance, with a sharp increase after the initial trials, being the second-best strategy. Grid search and random search demonstrate a more gradual and stepwise improvement, reflecting their exhaustive nature, eventually approaching competitive performance but requiring more evaluations to do so, still being lower than Bayesian-based strategies. Overall, HEPO was the best hypertuning strategy, and therefore, it is used in this paper.

### Cross-validation study

Table 1 presents an evaluation of the performance of the proposed stacked GAT model under varying numbers of nearest neighbors (N) and cross-validation folds. Our model demonstrates high classification performance, with accuracy values consistently above 0.90 across different configurations. This confirms the model’s ability to effectively exploit graph-structured data and capture node-level dependencies through the attention mechanism. In particular, the adaptive weighting of neighbors through the attention coefficients enables the GAT to dynamically focus on the most informative nodes, which appears to be especially effective in configurations with moderate neighbor counts.

**Table 1:** Cross-validation split and nearest neighbors analysis.

N	Folds	Accuracy	Precision	Recall	F1-score
3	5	*0.9533 ± 0.0933*	**1.0000** ± 0.0000	*0.8600 ± 0.2800*	*0.9247 ± 0.2154*
	10	*0.9467 ± 0.1108*	*0.9500 ± 0.1500*	*0.9400 ± 0.1800*	*0.9450 ± 0.1539*
	15	*0.9800 ± 0.0542*	*0.9867 ± 0.0499*	*0.9556 ± 0.1663*	*0.9709 ± 0.1258*
	20	*0.9866 ± 0.0403*	*0.9833 ± 0.0726*	**0.9833** ± 0.0726	*0.9833 ± 0.0600*
	25	*0.9333 ± 0.1700*	*0.8960 ± 0.2565*	*0.9600 ± 0.1960*	*0.9269 ± 0.2274*
5	5	*0.8800 ± 0.1694*	*0.8870 ± 0.2261*	*0.9000 ± 0.2000*	*0.8934 ± 0.1792*
	10	*0.9533 ± 0.0733*	*0.9339 ± 0.1336*	*0.9600 ± 0.1200*	*0.9468 ± 0.1008*
	15	**0.9933** ± 0.0249	*1.0000 ± 0.0000*	*0.9778 ± 0.0831*	**0.9888** ± 0.0499
	20	*0.9625 ± 0.1375*	*0.9688 ± 0.1362*	**0.9833** ± 0.0726	*0.9760 ± 0.1061*
	25	*0.9867 ± 0.0653*	*0.9600 ± 0.1960*	*0.9600 ± 0.1960*	*0.9600 ± 0.1960*
7	5	*0.9000 ± 0.1333*	*0.9000 ± 0.2000*	*0.9000 ± 0.2000*	*0.9000 ± 0.1633*
	10	*0.9667 ± 0.1000*	*0.9000 ± 0.3000*	*0.9000 ± 0.3000*	*0.9000 ± 0.3000*
	15	*0.9467 ± 0.1543*	*0.9600 ± 0.1497*	*0.9556 ± 0.1663*	*0.9578 ± 0.1584*
	20	*0.9616 ± 0.0985*	*0.9333 ± 0.2261*	*0.9167 ± 0.2555*	*0.9249 ± 0.2393*
	25	*0.9400 ± 0.1818*	*0.9467 ± 0.1809*	*0.9800 ± 0.0980*	*0.9630 ± 0.1470*
9	5	*0.9600 ± 0.0646*	*0.9333 ± 0.1333*	*0.9800 ± 0.0400*	*0.9561 ± 0.0775*
	10	*0.9333 ± 0.1333*	*0.8500 ± 0.3202*	*0.9000 ± 0.3000*	*0.8743 ± 0.3055*
	15	*0.9733 ± 0.0772*	*0.9714 ± 0.1069*	*0.9778 ± 0.0831*	*0.9746 ± 0.0814*
	20	*0.9875 ± 0.0545*	**1.0000** ± 0.0000	*0.9667 ± 0.1453*	*0.9831 ± 0.1090*
	25	*0.9800 ± 0.0718*	*0.9600 ± 0.1960*	*0.9400 ± 0.2154*	*0.9499 ± 0.2040*

Best results in bold.

With 3 neighbors, the model performs remarkably well, especially with 15 and 20 folds, achieving up to 98.66% accuracy and balanced metrics. However, the high variance in the 5-fold setting suggests some instability or overfitting in smaller training sets. With 5 neighbors, the GAT achieves its best performance, with a peak accuracy of 99.33% and an F1-score of 0.9888 at 15 folds. This configuration appears to optimally balance the local and contextual information, highlighting the GAT’s strength in aggregating relevant node features without over-smoothing.

With 7 neighbors, results remain strong but show slightly higher variance, particularly in the 10-fold and 25-fold settings. Precision and recall fluctuate more, possibly indicating diminishing returns from additional neighbors beyond the optimal range. With 9 neighbors, the model still performs well, but some degradation is observed in precision (e.g., *0.8500 ± 0.3202* in 10 folds), which could point to increased noise or over-smoothing effects as the neighborhood becomes too large.

Increasing the number of folds showed reduced variance and improved generalization, particularly noticeable in the transitions from 5 to 15 or 20 folds. The best-performing configurations (15-fold with 5 neighbors) benefit from more stable training-test splits, allowing the model to refine its attention mechanisms more effectively.

### Statistical analysis

The statistical analysis aims to evaluate the variability and robustness of the proposed model, considering several experiments conducted using a random initialization of the model weights.

#### Performance over 50 runs

**Fig. 7:**
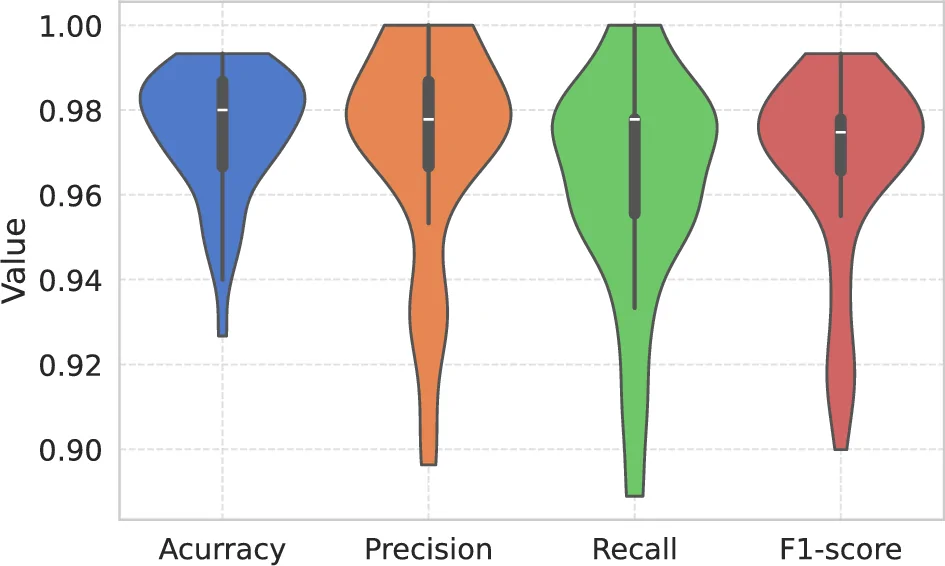
Violin plot of performances over 50 runs.

Considering the importance of generalization and stability, 50 experiments were conducted to assess whether our model stays stable given random initializations of the network weights. Figure 7 shows a violin plot to provide a visualization of the distribution and density of values for the performance metrics across multiple runs.

From Fig. 7, it is evident that all four metrics are centered at high values, above 0.96, reflecting strong model performance. However, differences in the distribution width and tail behavior are noteworthy. Precision and recall exhibit broader distributions compared to Accuracy, indicating higher variability in those metrics. Precision, in particular, displays a bimodal-like distribution, suggesting that models perform very well, but sometimes fall significantly short, potentially due to variability in false positive rates.

#### Empirical cumulative distribution analysis

**Fig. 8:**
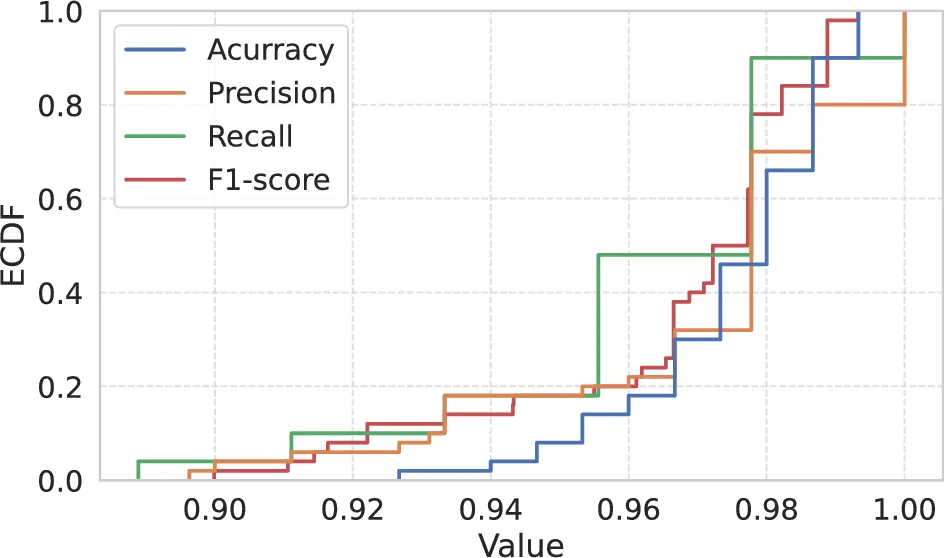
ECDF results over 50 runs.

Recall shows a slightly wider lower tail than F1-score or Accuracy, which is consistent with the Empirical Cumulative Distribution Function (ECDF) results (see Fig. 8), suggesting that although recall often reaches high values, it is more sensitive to fluctuations, possibly caused by missed positive samples. F1-score, being a harmonic mean of Precision and Recall, exhibits a shape that reflects both sources of variation but appears more centered and less skewed, indicating it may be a more stable summary metric in this context. Figure 8 shows the ECDF plot illustrating the cumulative distribution of performance metrics across the experiments.

From the ECDF curves, the Recall metric exhibits a steep ascent, indicating that the majority of the recall values are concentrated in the upper quantiles (above 0.96). This suggests that the models consistently achieved high recall values, with limited dispersion. In contrast, the Precision and Accuracy distributions demonstrate a more gradual slope, particularly in the lower portion of the curve, suggesting greater variability and a broader distribution of values across experiments.

Considering the high average values of the performance metrics and that the dispersion is possibly caused by false positives (synthetically generated), the results validate the suitability of the proposed stacked GAT for tasks requiring node-level predictions with complex interdependencies, and they highlight the importance of careful tuning of hyperparameters. Based on this validation of the model, a comparative analysis is presented in the next subsection.

### Benchmarking

For the benchmarking, we compare our method (stacked GAT hypertuned by HEPO) to the K-NN, RF, Naive Bayes, Logistic Regression (LR), Gradient Boosting (GradBoost), Adaptive Boosting (AdaBoost), Light Gradient Boosting Machine (LightGBM), Extreme Gradient Boosting (XGBoost), GCN, GAT, and Graph Sample and Aggregation Network (GraphSAGE).

The classifiers compared were used with their default settings, based on the sklearn library for tabular data models and torch_geometric.nn for graph-based models. In this study, the analyses are conducted with a small number of samples, which is a problem encountered in this evaluation.

#### Number of samples evaluation

Table 2 presents an analysis of the application of different classifiers using 10 samples with 5 features. The evaluation results highlight the superior performance of our model, which achieved the highest accuracy (*0.9667 ± 0.0667*), precision (*1.0000 ± 0.0000*), recall (*0.9000 ± 0.2000*), and F1-score (*0.9474 ± 0.1333*) among all methods.

**Table 2:** Experiment using 10 samples.

Model	Accuracy	Precision	Recall	F1-score
MLP	*0.3333 ± 0.2981*	*0.2444 ± 0.2037*	*0.3333 ± 0.2981*	*0.2821 ± 0.2400*
K-NN	*0.6667 ± 0.0000*	*0.4444 ± 0.0000*	*0.6667 ± 0.0000*	*0.5333 ± 0.0000*
SVM	*0.6667 ± 0.0000*	*0.4444 ± 0.0000*	*0.6667 ± 0.0000*	*0.5333 ± 0.0000*
RF	*0.6000 ± 0.2494*	*0.5111 ± 0.2494*	*0.6000 ± 0.2494*	*0.5520 ± 0.2437*
Naive Bayes	*0.5333 ± 0.1633*	*0.5444 ± 0.3538*	*0.5333 ± 0.1633*	*0.5388 ± 0.2449*
LR	*0.8000 ± 0.1633*	*0.6667 ± 0.2722*	*0.8000 ± 0.1633*	*0.7273 ± 0.2286*
GradBoost	*0.5333 ± 0.3399*	*0.4444 ± 0.3220*	*0.5333 ± 0.3399*	*0.4848 ± 0.3250*
AdaBoost	*0.6000 ± 0.2494*	*0.5111 ± 0.2494*	*0.6000 ± 0.2494*	*0.5520 ± 0.2437*
LightGBM	*0.6667 ± 0.0000*	*0.4444 ± 0.0000*	*0.6667 ± 0.0000*	*0.5333 ± 0.0000*
XGBoost	*0.6000 ± 0.1333*	*0.4222 ± 0.0444*	*0.6000 ± 0.1333*	*0.4957 ± 0.0800*
GNC	*0.8000 ± 0.2667*	*0.6000 ± 0.4899*	*0.6000 ± 0.4899*	*0.6000 ± 0.4899*
GAT	*0.8667 ± 0.1633*	*0.6000 ± 0.4899*	*0.6000 ± 0.4899*	*0.6000 ± 0.4899*
GraphSAGE	*0.8667 ± 0.1633*	*0.6000 ± 0.4899*	*0.6000 ± 0.4899*	*0.6000 ± 0.4899*
Ours	**0.9667** ± 0.0667	**1.0000** ± 0.0000	**0.9000** ± 0.2000	**0.9474** ± 0.1333

Best results in bold.

In this experiment, traditional models like LR (accuracy of 0.8000 ± 0.1633, F1-score of *0.7273 ± 0.2286*) and ensemble methods such as RF, AdaBoost, and XGBoost showed moderate performance, while deep learning-based graph models like GAT and GraphSAGE reached competitive accuracy levels (*0.8667 ± 0.1633*), though with greater variability and lower precision compared to the proposed approach. Simpler models like MLP and Naive Bayes underperformed, with MLP achieving the lowest accuracy (*0.3333 ± 0.2981*).

Table 3 presents an analysis considering the use of 15 samples with 5 characteristics each. With the increase to 15 training samples, overall model performance significantly improved across most methods. The proposed model maintained outstanding results, achieving an accuracy of *0.9556 ± 0.0889* and perfect precision (*1.0000 ± 0.0000*), while also attaining a high recall (*0.8667 ± 0.2667*) and F1-score (*0.9286 ± 0.2000*), indicating strong generalization and reliability. The GNC model slightly outperformed in terms of overall accuracy (*0.9778 ± 0.0444*) and also reached perfect precision, though its F1-score (*0.9655 ± 0.0800*) suggests slightly less variability in recall.

**Table 3:** Experiment using 15 samples.

Model	Accuracy	Precision	Recall	F1-score
MLP	*0.5333 ± 0.1633*	*0.5557 ± 0.1917*	*0.5333 ± 0.1633*	*0.5443 ± 0.1712*
K-NN	*0.8444 ± 0.1133*	*0.8478 ± 0.1246*	*0.8444 ± 0.1133*	*0.8461 ± 0.1196*
SVM	*0.8889 ± 0.0703*	*0.9109 ± 0.0530*	*0.8889 ± 0.0703*	*0.8998 ± 0.0833*
RF	*0.9111 ± 0.0831*	*0.9294 ± 0.0636*	**0.9111** ± 0.0831	*0.9202 ± 0.0970*
Naive Bayes	*0.6222 ± 0.0889*	*0.7997 ± 0.0848*	*0.6222 ± 0.0889*	*0.6999 ± 0.0772*
LR	*0.8889 ± 0.0994*	*0.9044 ± 0.0888*	*0.8889 ± 0.0994*	*0.8966 ± 0.1091*
GradBoost	*0.8667 ± 0.0831*	*0.8991 ± 0.0580*	*0.8667 ± 0.0831*	*0.8826 ± 0.0926*
AdaBoost	*0.8667 ± 0.0831*	*0.8991 ± 0.0580*	*0.8667 ± 0.0831*	*0.8826 ± 0.0926*
LightGBM	*0.6667 ± 0.0994*	*0.4543 ± 0.1326*	*0.6667 ± 0.0994*	*0.5404 ± 0.1269*
XGBoost	*0.8889 ± 0.0703*	*0.9102 ± 0.0530*	*0.8889 ± 0.0703*	*0.8994 ± 0.0832*
GNC	*0.9778 ± 0.0444*	*1.0000 ± 0.0000*	*0.9333 ± 0.1333*	*0.9655 ± 0.0800*
GAT	*0.9111 ± 0.1296*	*0.7500 ± 0.3873*	*0.8000 ± 0.4000*	*0.7742 ± 0.3897*
GraphSAGE	*0.8889 ± 0.1721*	*0.8167 ± 0.2603*	*0.8667 ± 0.2667*	*0.8409 ± 0.2584*
Ours	**0.9556** ± 0.0889	**1.0000** ± 0.0000	*0.8667 ± 0.2667*	**0.9286** ± 0.2000

Best results in bold.

Classical methods like RF, SVM, LR, and XGBoost also performed strongly, with F1-scores above 0.88, reflecting their stability with more data. In contrast, models like MLP and LightGBM continued to show lower effectiveness, particularly in precision and F1-score. Notably, graph-based models such as GAT and GraphSAGE displayed high variance in performance, likely due to their sensitivity to small datasets, although GraphSAGE achieved an F1-score of 0.8409 ± 0.2584. These results confirm that increasing the sample size boosts model robustness, and that the proposed model remains among the top performers in terms of balanced classification metrics.

Table 4 shows the performance results obtained using 20 samples with 5 features. With the training set expanded to 20 samples, most models exhibited enhanced stability and performance. The proposed model achieved an accuracy of *0.9333 ± 0.0972,* with high precision (*0.9143 ± 0.1714*), recall (*0.9500 ± 0.1000*), and F1-score (*0.9318 ± 0.1098*), indicating a strong balance between correct predictions and consistency across folds. Similarly, RF matched this accuracy with slightly better F1-score stability (*0.9373 ± 0.0345*), showcasing its robustness with small datasets.

**Table 4:** Experiment using 20 samples.

Model	Accuracy	Precision	Recall	F1-score
MLP	*0.5833 ± 0.1179*	*0.5772 ± 0.1728*	*0.5833 ± 0.1179*	*0.5802 ± 0.1516*
K-NN	*0.9000 ± 0.1225*	*0.9148 ± 0.1018*	*0.9000 ± 0.1225*	*0.9073 ± 0.1187*
SVM	*0.9167 ± 0.0913*	*0.9247 ± 0.0835*	*0.9167 ± 0.0913*	*0.9206 ± 0.0893*
RF	**0.9333** ± 0.0333	*0.9413 ± 0.0294*	*0.9333 ± 0.0333*	*0.9373 ± 0.0345*
Naive Bayes	*0.6333 ± 0.1546*	*0.8437 ± 0.0356*	*0.6333 ± 0.1546*	*0.7235 ± 0.1761*
LR	*0.9167 ± 0.0913*	**0.9247** ± 0.0835	*0.9167 ± 0.0913*	*0.9206 ± 0.0893*
GradBoost	*0.8833 ± 0.0667*	*0.9002 ± 0.0581*	*0.8833 ± 0.0667*	*0.8917 ± 0.0701*
AdaBoost	*0.8667 ± 0.0850*	*0.8766 ± 0.0823*	*0.8667 ± 0.0850*	*0.8716 ± 0.0868*
LightGBM	*0.8833 ± 0.0667*	*0.9069 ± 0.0515*	*0.8833 ± 0.0667*	*0.8949 ± 0.0728*
XGBoost	*0.8500 ± 0.0624*	*0.8987 ± 0.0248*	*0.8500 ± 0.0624*	*0.8737 ± 0.0536*
GNC	**0.9333** ± 0.0816	*0.8833 ± 0.1453*	**0.9500** ± 0.1000	*0.9155 ± 0.1114*
GAT	*0.8667 ± 0.1633*	*0.8000 ± 0.2449*	*0.9000 ± 0.2000*	*0.8471 ± 0.2108*
GraphSAGE	*0.8833 ± 0.2333*	*0.8400 ± 0.3200*	*0.8500 ± 0.3000*	*0.8450 ± 0.3111*
Ours	**0.9333** ± 0.0972	*0.9143 ± 0.1714*	**0.9500** ± 0.1000	**0.9318** ± 0.1098

Best results in bold.

Traditional models like SVM and LR performed nearly identically (accuracy and F1-score around 0.92), reinforcing their reliability. K-NN, GradBoost, LightGBM, and AdaBoost also maintained high effectiveness, all with F1-scores above 0.87. Meanwhile, GNC stood out with the highest recall (*0.9500 ± 0.1000*), although its precision showed more variance. Graph-based models like GAT and GraphSAGE showed decent results but with higher variability, reflecting their sensitivity to small sample sizes. In contrast, MLP and Naive Bayes lagged behind in performance, particularly in recall and F1-score. Overall, the results confirm that with 20 samples, most models reach a high level of accuracy and consistency, with the proposed method remaining among the top performers.

The results in Table 5 indicate a clear performance differentiation between traditional machine learning models, graph-based approaches, and the proposed method with 30 samples. Our model, Naive Bayes and XGBoost, reach the highest accuracy of *0.9778 ± 0.0272*, followed closely by ensemble methods such as RF, GradBoost, AdaBoost, and LightGBM, all achieving approximately 0.9667. These results indicate that, for larger datasets, tabular machine learning models are satisfactory to classify the sensor as faulty.

**Table 5:** Experiment using 30 samples.

Model	Accuracy	Precision	Recall	F1-score
MLP	*0.7111 ± 0.0956*	*0.7299 ± 0.2123*	*0.7111 ± 0.0956*	*0.7204 ± 0.1429*
KNN	*0.9222 ± 0.0272*	*0.9318 ± 0.0205*	*0.9222 ± 0.0272*	*0.9270 ± 0.0279*
SVM	*0.9222 ± 0.0272*	*0.9318 ± 0.0205*	*0.9222 ± 0.0272*	*0.9270 ± 0.0279*
RF	*0.9667 ± 0.0272*	*0.9695 ± 0.0249*	*0.9667 ± 0.0272*	*0.9681 ± 0.0276*
Naive Bayes	**0.9778** ± 0.0272	*0.9796 ± 0.0249*	**0.9778** ± 0.0272	**0.9787** ± 0.0277
LR	*0.9444 ± 0.0351*	*0.9505 ± 0.0298*	*0.9444 ± 0.0351*	*0.9475 ± 0.0366*
GradBoost	*0.9667 ± 0.0272*	*0.9695 ± 0.0249*	*0.9667 ± 0.0272*	*0.9681 ± 0.0276*
AdaBoost	*0.9667 ± 0.0272*	*0.9695 ± 0.0249*	*0.9667 ± 0.0272*	*0.9681 ± 0.0276*
LightGBM	*0.9667 ± 0.0444*	*0.9676 ± 0.0440*	*0.9667 ± 0.0444*	*0.9671 ± 0.0445*
XGBoost	**0.9778** ± 0.0272	*0.9796 ± 0.0249*	**0.9778** ± 0.0272	*0.9787 ± 0.0277*
GNC	*0.9444 ± 0.1111*	**1.0000** ± 0.0000	*0.8333 ± 0.3333*	*0.9091 ± 0.2857*
GAT	*0.9333 ± 0.1077*	*0.9714 ± 0.0571*	*0.8333 ± 0.3333*	*0.8971 ± 0.2796*
GraphSAGE	*0.9222 ± 0.1089*	*0.9500 ± 0.1000*	*0.8333 ± 0.3333*	*0.8879 ± 0.2770*
Ours	**0.9778** ± 0.0272	*0.9714 ± 0.0571*	*0.9667 ± 0.0667*	*0.9690 ± 0.0413*

Best results in bold.

The proposed method achieves *0.9778 ± 0.0272* accuracy, matching the best performing models while providing a more balanced trade-off between precision $\left( 0.9714 \pm 0.0571\right)$ and recall $\left( 0.9667 \pm 0.0667\right)$. Consequently, it yields a strong F1-score of *0.9690 ± 0.0413*, indicating consistent classification performance across folds. Compared to other graph-based methods, the proposed approach significantly improves stability and generalization, reducing variance and mitigating the recall degradation observed in standard GNN architectures.

For larger datasets, the model remains competitive in terms of classification performance but requires greater computational effort. For specific conditions involving large datasets, it would be necessary to re-make hypertuning so that the model could capture the characteristics of the data distribution and thereby improve its performance. Given that the proposed model was designed for use with small datasets (less than 20 samples), the analysis focused on this scenario demonstrated that the proposed approach outperforms the other compared models.

### Generalization over several types of NPP faults

To further evaluate the proposed stacked GAT model, we compare our model to other models for five different types of NPP faults. Specifically, we consider: Table 6 for loss of AC power; Table 7 for steam generator A tube rupture; Table 8 for steam generator B tube rupture; Table 9 for steam line break inside containment; and Table 10 for the letdown line break in the auxiliary building.

The results in Table 6 present the performance of various models in diagnosing the loss of AC power fault in a NPP. Classical approaches such as LR and LightGBM showed strong and stable results, both reaching an accuracy of 0.9333 with balanced precision, recall, and F1-score values. Among ensemble methods, RF, GradBoost, AdaBoost, and XGBoost achieved competitive but slightly lower results, while Naive Bayes performed poorly, with accuracy dropping to 0.6333, highlighting its limitations for this fault scenario.

Graph-based models provided the best overall performance: GraphSAGE reached perfect recall with an accuracy of 0.9333, while GAT and the proposed method achieved the highest accuracies of 0.9500. Notably, the proposed method outperformed all others in terms of F1-score (0.9595) by combining high precision and perfect recall, ensuring reliable detection of this critical fault. These findings confirm the advantage of graph learning approaches, particularly the proposed method, for capturing the complex dependencies present in NPP fault data.

**Table 6:** Experiment of loss of AC power NPP fault.

Model	Accuracy	Precision	Recall	F1-score
MLP	*0.7667 ± 0.2494*	*0.7708 ± 0.2938*	*0.7667 ± 0.2494*	*0.7687 ± 0.2809*
K-NN	*0.9000 ± 0.1528*	*0.9042 ± 0.1606*	*0.9000 ± 0.1528*	*0.9021 ± 0.1649*
SVM	*0.9167 ± 0.1179*	*0.9083 ± 0.1506*	*0.9167 ± 0.1179*	*0.9125 ± 0.1450*
RF	*0.8833 ± 0.1546*	*0.9181 ± 0.1204*	*0.8833 ± 0.1546*	*0.9004 ± 0.1647*
Naive Bayes	*0.6333 ± 0.2014*	*0.7819 ± 0.2558*	*0.6333 ± 0.2014*	*0.6998 ± 0.2305*
LR	*0.9333 ± 0.1106*	*0.9375 ± 0.1200*	*0.9333 ± 0.1106*	*0.9354 ± 0.1295*
GradBoost	*0.8500 ± 0.2000*	*0.8583 ± 0.2427*	*0.8500 ± 0.2000*	*0.8541 ± 0.2360*
AdaBoost	*0.8667 ± 0.2211*	*0.8528 ± 0.2647*	*0.8667 ± 0.2211*	*0.8597 ± 0.2556*
LightGBM	*0.9333 ± 0.1106*	**0.9403** ± 0.1181	*0.9333 ± 0.1106*	*0.9368 ± 0.1265*
XGBoost	*0.8500 ± 0.2000*	*0.8736 ± 0.1867*	*0.8500 ± 0.2000*	*0.8616 ± 0.2054*
GNC	*0.9167 ± 0.1972*	*0.8167 ± 0.3704*	*0.8667 ± 0.3399*	*0.8409 ± 0.3568*
GAT	**0.9500** ± 0.1000	*0.8667 ± 0.2867*	*0.9333 ± 0.2494*	*0.8988 ± 0.2629*
GraphSAGE	*0.9333 ± 0.1434*	*0.8889 ± 0.2250*	**1.0000** ± 0.0000	*0.9412 ± 0.1595*
Ours	**0.9500** ± 0.1354	*0.9222 ± 0.2006*	**1.0000** ± 0.0000	**0.9595** ± 0.1449

Best results in bold.

The results in Table 7 report the performance of different models for the steam generator A tube rupture fault. Traditional machine learning methods such as K-NN, SVM, LR, GradBoost, AdaBoost, and XGBoost achieved consistent performance, with accuracies around 0.9167 to 0.9333 and balanced precision and recall. RF slightly outperformed them with 0.9500 accuracy and a stable F1-score. In contrast, MLP and Naive Bayes underperformed, with accuracies below 0.70, showing limitations in capturing the complexity of this fault.

Graph-based models clearly improved the results: GNC and GAT both reached 0.9667 accuracy, while GraphSAGE followed closely with 0.9500, though their precision and recall showed higher variance. The proposed method delivered the best overall performance with 0.9833 accuracy and a perfect recall of 1.0000, resulting in the highest F1-score of 0.9535. These findings highlight that while ensemble methods remain competitive, graph learning approaches, especially the proposed model, provide superior fault detection reliability for this scenario.

**Table 7:** Experiment of steam generator A tube rupture NPP fault.

Model	Accuracy	Precision	Recall	F1-score
MLP	*0.6833 ± 0.2134*	*0.6375 ± 0.2670*	*0.6833 ± 0.2134*	*0.6596 ± 0.2407*
K-NN	*0.9333 ± 0.1106*	*0.9375 ± 0.1200*	*0.9333 ± 0.1106*	*0.9354 ± 0.1295*
SVM	*0.9167 ± 0.1179*	*0.9083 ± 0.1506*	*0.9167 ± 0.1179*	*0.9125 ± 0.1450*
RF	*0.9500 ± 0.1000*	**0.9514** ± 0.1153	*0.9500 ± 0.1000*	*0.9507 ± 0.1167*
Naive Bayes	*0.6667 ± 0.1972*	*0.7694 ± 0.2629*	*0.6667 ± 0.1972*	*0.7144 ± 0.2286*
LR	*0.9333 ± 0.1106*	*0.9375 ± 0.1200*	*0.9333 ± 0.1106*	*0.9354 ± 0.1295*
GradBoost	*0.9333 ± 0.1434*	*0.9486 ± 0.1176*	*0.9333 ± 0.1434*	*0.9409 ± 0.1559*
AdaBoost	*0.9333 ± 0.1434*	*0.9486 ± 0.1176*	*0.9333 ± 0.1434*	*0.9409 ± 0.1559*
LightGBM	*0.9167 ± 0.1179*	*0.9139 ± 0.1485*	*0.9167 ± 0.1179*	*0.9153 ± 0.1403*
XGBoost	*0.9333 ± 0.1106*	**0.9514** ± 0.1153	*0.9333 ± 0.1106*	*0.9423 ± 0.1174*
GNC	*0.9667 ± 0.0850*	*0.9000 ± 0.2708*	*0.9333 ± 0.2494*	*0.9164 ± 0.2572*
GAT	*0.9667 ± 0.0850*	*0.9000 ± 0.2708*	*0.9333 ± 0.2494*	*0.9164 ± 0.2572*
GraphSAGE	*0.9500 ± 0.1354*	*0.8889 ± 0.2897*	*0.9333 ± 0.2494*	*0.9106 ± 0.2708*
Ours	**0.9833** ± 0.0624	*0.9111 ± 0.2266*	**1.0000** ± 0.0000	**0.9535** ± 0.1700

Best results in bold.

The results in Table 8 present the evaluation of different models for detecting the steam generator B tube rupture fault. Conventional models such as LR, GradBoost, RF, and AdaBoost demonstrated strong and consistent performance, with accuracies between 0.9333 and 0.9500, supported by balanced precision, recall, and F1-scores. Ensemble methods, particularly AdaBoost, achieved one of the best results among classical approaches, with an accuracy of 0.9500 and an F1-score of 0.9507. In contrast, MLP and Naive Bayes underperformed, with accuracies of 0.4833 and 0.6500, respectively, reflecting difficulty in handling this fault pattern.

Graph-based models achieved competitive performance, with GNC, GAT, and GraphSAGE reaching accuracies between 0.9333 and 0.9500, although their precision and recall values showed higher variability. The proposed method significantly outperformed all baselines, achieving the highest accuracy of 0.9833, with stable precision, recall, and F1-score values, confirming its superior ability to capture the complex relationships in the data. These results emphasize that while ensemble learning remains strong, the proposed graph-based model delivers the most reliable and accurate detection for this fault scenario.

**Table 8:** Experiment of steam generator B tube rupture NPP fault.

Model	Accuracy	Precision	Recall	F1-score
MLP	*0.4833 ± 0.2656*	*0.4444 ± 0.3227*	*0.4833 ± 0.2656*	*0.4631 ± 0.2777*
K-NN	*0.9167 ± 0.1179*	*0.9083 ± 0.1506*	*0.9167 ± 0.1179*	*0.9125 ± 0.1450*
SVM	*0.9167 ± 0.1179*	*0.9083 ± 0.1506*	*0.9167 ± 0.1179*	*0.9125 ± 0.1450*
RF	*0.9333 ± 0.1434*	*0.9486 ± 0.1176*	*0.9333 ± 0.1434*	*0.9409 ± 0.1559*
Naive Bayes	*0.6500 ± 0.2198*	*0.7639 ± 0.2745*	*0.6500 ± 0.2198*	*0.7024 ± 0.2430*
LR	*0.9333 ± 0.1106*	*0.9375 ± 0.1200*	*0.9333 ± 0.1106*	*0.9354 ± 0.1295*
GradBoost	*0.9333 ± 0.1434*	*0.9486 ± 0.1176*	*0.9333 ± 0.1434*	*0.9409 ± 0.1559*
AdaBoost	*0.9500 ± 0.1000*	**0.9514** ± 0.1153	**0.9500** ± 0.1000	**0.9507** ± 0.1167
LightGBM	*0.9167 ± 0.1491*	*0.9403 ± 0.1181*	*0.9167 ± 0.1491*	*0.9283 ± 0.1592*
XGBoost	*0.9167 ± 0.1491*	*0.9486 ± 0.1176*	*0.9167 ± 0.1491*	*0.9324 ± 0.1554*
GNC	*0.9333 ± 0.1929*	*0.8833 ± 0.3009*	*0.9333 ± 0.2494*	*0.9076 ± 0.2816*
GAT	*0.9500 ± 0.1354*	*0.8889 ± 0.2897*	*0.9333 ± 0.2494*	*0.9106 ± 0.2708*
GraphSAGE	*0.9333 ± 0.1929*	*0.8833 ± 0.3009*	*0.9333 ± 0.2494*	*0.9076 ± 0.2816*
Ours	**0.9833** ± 0.0624	*0.9333 ± 0.2494*	*0.9333 ± 0.2494*	*0.9333 ± 0.2494*

Best results in bold.

The results in Table 9 show the comparative performance for a steam line break inside containment. Traditional models such as Naive Bayes and XGBoost achieved strong performance, both reaching 0.9667 accuracy with balanced precision, recall, and F1-score values, indicating reliable detection capabilities. LightGBM and LR also performed consistently with accuracy levels around 0.9333.

Among graph-based models, the GAT and the proposed method (Ours) achieved the highest accuracy of 0.9833, surpassing all baselines, although their precision and recall exhibited higher variance compared to ensemble methods, reflecting sensitivity to data variability. Overall, the findings highlight that while ensemble learning remains highly competitive, graph neural networks, particularly GAT and the proposed approach, deliver state-of-the-art accuracy, making them promising solutions for fault diagnosis in NPP safety systems.

**Table 9:** Experiment of steam line break inside containment NPP fault.

Model	Accuracy	Precision	Recall	F1-score
MLP	*0.7167 ± 0.2718*	*0.7208 ± 0.2963*	*0.7167 ± 0.2718*	*0.7187 ± 0.2836*
K-NN	*0.9333 ± 0.1106*	*0.9375 ± 0.1200*	*0.9333 ± 0.1106*	*0.9354 ± 0.1295*
SVM	*0.9333 ± 0.1106*	*0.9375 ± 0.1200*	*0.9333 ± 0.1106*	*0.9354 ± 0.1295*
RF	*0.9167 ± 0.1491*	*0.9375 ± 0.1200*	*0.9167 ± 0.1491*	*0.9270 ± 0.1614*
Naive Bayes	*0.9667 ± 0.0850*	*0.9597 ± 0.1140*	*0.9667 ± 0.0850*	*0.9632 ± 0.1073*
LR	*0.9333 ± 0.1106*	*0.9375 ± 0.1200*	*0.9333 ± 0.1106*	*0.9354 ± 0.1295*
GradBoost	*0.9167 ± 0.1491*	*0.9375 ± 0.1200*	*0.9167 ± 0.1491*	*0.9270 ± 0.1614*
AdaBoost	*0.9167 ± 0.1491*	*0.9375 ± 0.1200*	*0.9167 ± 0.1491*	*0.9270 ± 0.1614*
LightGBM	*0.9333 ± 0.1434*	*0.9486 ± 0.1176*	*0.9333 ± 0.1434*	*0.9409 ± 0.1559*
XGBoost	*0.9667 ± 0.0850*	**0.9597** ± 0.1140	**0.9667** ± 0.0850	**0.9632** ± 0.1073
GNC	*0.9667 ± 0.0850*	*0.8667 ± 0.3399*	*0.8667 ± 0.3399*	*0.8667 ± 0.3399*
GAT	*0.9833 ± 0.0624*	*0.9333 ± 0.2494*	*0.9333 ± 0.2494*	*0.9333 ± 0.2494*
GraphSAGE	*0.9667 ± 0.0850*	*0.8667 ± 0.3399*	*0.8667 ± 0.3399*	*0.8667 ± 0.3399*
Ours	**0.9833** ± 0.0624	*0.9333 ± 0.2494*	*0.9333 ± 0.2494*	*0.9333 ± 0.2494*

Best results in bold.

The results in Table 10 summarize the performance of different models for detecting the letdown line break fault in the auxiliary building. Traditional methods showed heterogeneous behavior: K-NN achieved the best accuracy among them with 0.8500 and a stable F1-score of 0.8521, while RF, SVM, GradBoost, and XGBoost provided competitive but slightly lower results. Simpler models such as MLP, LR, and Naive Bayes performed poorly, with accuracies below 0.65, indicating difficulty in generalizing this fault. Ensemble methods like AdaBoost and LightGBM also underperformed, showing instability across precision and recall.

Graph-based approaches presented mixed results: GNC and GAT achieved moderate performance but with high variability, reflecting sensitivity to data imbalance. GraphSAGE improved stability, reaching 0.8333 accuracy with good recall. The proposed method outperformed all baselines, reaching 0.9000 accuracy, the highest recall among graph-based models, and a balanced F1-score of 0.7611 despite variance in precision. These results confirm that while classical methods like K-NN remain strong, the proposed graph-based approach provides superior fault detection capability in this challenging scenario.

**Table 10:** Experiment of letdown line break in auxiliary building NPP fault.

Model	Accuracy	Precision	Recall	F1-score
MLP	*0.5333 ± 0.2560*	*0.5222 ± 0.2884*	*0.5333 ± 0.2560*	*0.5277 ± 0.2495*
K-NN	*0.8500 ± 0.1225*	*0.8542 ± 0.1603*	*0.8500 ± 0.1225*	*0.8521 ± 0.1457*
SVM	*0.8167 ± 0.1434*	*0.7986 ± 0.1803*	*0.8167 ± 0.1434*	*0.8075 ± 0.1679*
RF	*0.8167 ± 0.2494*	**0.8139** ± 0.2607	*0.8167 ± 0.2494*	**0.8153** ± 0.2568
Naive Bayes	*0.6333 ± 0.1795*	*0.6778 ± 0.2012*	*0.6333 ± 0.1795*	*0.6548 ± 0.1526*
LR	*0.5667 ± 0.1700*	*0.4528 ± 0.2200*	*0.5667 ± 0.1700*	*0.5034 ± 0.1935*
GradBoost	*0.7833 ± 0.2560*	*0.7333 ± 0.3338*	*0.7833 ± 0.2560*	*0.7575 ± 0.3035*
AdaBoost	*0.6500 ± 0.2858*	*0.6458 ± 0.3276*	*0.6500 ± 0.2858*	*0.6479 ± 0.3017*
LightGBM	*0.6667 ± 0.2528*	*0.6597 ± 0.3172*	*0.6667 ± 0.2528*	*0.6632 ± 0.2828*
XGBoost	*0.7833 ± 0.2718*	*0.7944 ± 0.3036*	*0.7833 ± 0.2718*	*0.7888 ± 0.2828*
GNC	*0.7167 ± 0.2867*	*0.5389 ± 0.4035*	*0.8000 ± 0.4000*	*0.6440 ± 0.3811*
GAT	*0.7000 ± 0.2915*	*0.5278 ± 0.4067*	*0.8000 ± 0.4000*	*0.6360 ± 0.3815*
GraphSAGE	*0.8333 ± 0.2357*	*0.6944 ± 0.3917*	**0.8667** ± 0.3399	*0.7711 ± 0.3596*
Ours	**0.9000** ± 0.1528	*0.7556 ± 0.4122*	*0.7667 ± 0.4028*	*0.7611 ± 0.3985*

Best results in bold.

Across all evaluated fault scenarios, the results demonstrate that classical machine learning methods such as K-NN, SVM, LR, and ensemble models (RF, GradBoost, AdaBoost, LightGBM, and XGBoost) generally achieved strong and stable performance, often surpassing 0.90 accuracy, with RF and AdaBoost consistently ranking among the best baselines. Simpler models like MLP and Naive Bayes frequently underperformed, showing limited ability to capture the complexity of NPP fault patterns.

Overall, graph-based learning approaches provided superior results in most cases, with GAT, GraphSAGE, and GNC achieving competitive performance but with higher variability. The proposed method consistently outperformed all others, reaching the highest accuracy values (up to 0.9833) and maintaining strong recall, often achieving perfect fault detection in several scenarios. Overall, the findings confirm that while ensemble methods remain reliable baselines, graph-based learning, and particularly the proposed model, offer the most robust and promising approach for NPP fault diagnosis Comparatively, Zhang et al.^61^ show that Bayesian theory is indeed promising for solving problems involving useful life prediction. Zhang et al.^62^ demonstrate that graph-based models can outperform well-established models such as CNNs and LSTMs, proving that the graph-based approach presented here can be applied to other challenges. Other techniques can also be used in this context, such as ensemble learning methods^63^, adaptation methods^64^, and variational autoencoder^65^, demonstrating that problems in the domain of fault diagnosis can be solved using a variety of methods.

## Final remarks

This work introduced a novel deep learning-based approach for fault identification in NPPs, focusing specifically on transient events and sensor anomalies. The methodology is centered on a stacked GAT architecture, which was optimized using an HEBO strategy. This combination allowed for effective modeling of complex interdependencies between system components by transforming transient operational data into graph representations. Each node in the graph corresponded to a system variable, while the edges captured the physical relationships inherent in NPP operations. The simulated dataset, generated using the PCTRAN, included fault conditions such as loss-of-coolant accident, one of the most critical issues faced in NPPs.

The proposed method achieved outstanding results across a wide range of configurations and evaluation metrics. In low-data scenarios, such as when using only 10 samples and 5 features, the model reached an accuracy of 96.67%, with a precision score of 100%, a recall of 90.00%, and an F1-score of 94.74%. As the number of training samples increased to 20, the model maintained promising performance.

Over 50 independent runs, the model consistently delivered high scores, with all key metrics centered above 0.96. Violin plots and ECDF analyses confirmed the model’s stability, showing low dispersion in accuracy, precision, recall, and F1-score. The model consistently outperformed classical machine learning methods such as SVM, RF, LR, and XGBoost, as well as deep learning models, including MLP, GraphSAGE, and standard GAT configurations. These results underline the superior capacity of the proposed model to handle both limited data scenarios and complex, high-dimensional feature spaces.

The results presented in this study are based on synthetic data generated using the PCTRAN simulator, which provides a controlled and reproducible environment for evaluating fault identification methods. While the proposed model demonstrates strong performance under these conditions, real nuclear power plant environments introduce additional challenges, including measurement noise, missing data, temporal misalignment, non-stationarity, and domain shifts across different reactor configurations.

Future work can be done to extend the current research in several meaningful directions, aiming to bridge the gap between high-performing algorithms and real-world applicability in NPP environments. The proposed model needs to be validated using real operational data, which typically contains noise, missing values, and unpredictable dynamics that are not fully captured in synthetic simulations. The transfer of models trained on simulated data to real-world industrial plant environments remains a significant challenge, and this area represents a future research direction.

The current architecture, while highly accurate, operates as a black-box system. To address this, future studies could incorporate explainable AI techniques, such as attention weight interpretation, feature relevance mapping, or interpretable surrogate models, to enhance transparency and foster greater trust among plant operators and engineers.

In this study, the focus was on distinguishing actual NPP faults from sensor-induced anomalies that may generate false fault alarms. The proposed approach does not identify the specific type of sensor fault. Future work may investigate multi-class classification approaches to distinguish among different sensor fault types and fault severities, providing a more comprehensive diagnostic framework.

## Data Availability

For future comparisons, the dataset is available at: https://github.com/SFStefenon/NPPmalfunctions.

## A PCTRAN saved variables

The evaluated malfunction was analyzed using the IAEA PCTRAN variables presented in Tables 11 and 12. These variables describe the main plant responses, including reactor power, coolant pressure and temperature, steam generator behavior, containment conditions, radiation monitors, emergency core cooling flows, and core damage indicators.

**Table 11:** IAEA PCTRAN variables (1/2).

Description	Acronym
Activity reactor coolant (CPM)	RC87
Clad failure (%)	FRCL
Concentration reactor building hydrogen (%)	CNH2
Concentration reactor coolant I-131 (GBq/cc)	RC131
Concentration reactor coolant system boron (ppm)	PPM
Dose rate exclusion area boundary thyroid (mSv/hr)	DTHY
Dose rate exclusion area boundary whole body (mSv/hr)	DWB
Flow accumulator (t/hr)	WCFT
Flow charging (t/hr)	WCHG
Flow containment spray (t/hr)	WCSP
Flow high pressure injection (t/hr)	WHPI
Flow letdown (t/hr)	WLD
Flow low-pressure injection (residual heat removal) (t/hr)	WLPI
Flow pressurizer spray (t/hr)	WSPY
Flow Przr power-operated relief valve and safeties (t/hr)	WUP
Flow reactor coolant system leak (t/hr)	WLR
Flow reactor coolant loop (t/hr)	WRCA and WRCB
Flow steam generator feedwater (t/hr)	WFWA and WFWB
Flow steam generator main steam valve/automatic depressurization system (t/hr)	WRLA and WRLB
Flow steam generator steam (t/hr)	WSTA and WSTB
Flow steam generator tube leak (t/hr)	WTRA and WTRB
Flow total break entering reactor building(t/hr)	WBK
Flow total emergency core cooling system (t/hr)	WECS
Fraction Zr oxidation	FRZR
Integrated break energy (MJ)	EBK
Integrated break flow (kg)	MBK
Level core water (m)	LVCR
Level pressurizer (%)	LVPZ
Level reactor building sump water (m)	LWRB
Level steam generator narrow range (%)	NSGA and NSGB
Level steam generator wide range (m)	LSGA and LSGB
Mass $\hbox {H}_2$ generated by Zr-$\hbox {H}_2$O (kg)	MH2
Mass of contingency cooling injection gases (kg)	MGAS
Mass of corium in DW (kg)	MDBR
Mass of molten concrete (kg)	MCRT
Mass total leakage out of reactor building(kg)	RBLK
Mass total leakage out of SGs (kg)	SGLK
Power core thermal (%)	PWR
Power fan cooler heat removal (MW)	QFCL
Power nuclear flux (%)	PWNT
Power pressurizer heater (kW)	HTR

**Table 12:** IAEA PCTRAN variables (2/2).

Description	Acronym
Power residual heat removal rate (MW)	QRHR
Power steam generator heat removal (MW)	QMGA and QMGB
Power total megawatt thermal (MW)	QMWT
Power turbine load (%)	TBLD
Press partial reactor building air (bar)	PRBA
Press reactor building (bar)	PRB
Press reactor coolant system (bar)	P
Pressure steam generator (bar)	PSGA and PSGB
Rad monitor aux building air (CPM)	RM4
Rad monitor condenser off-gas (CPM)	RM3
Rad monitor reactor building air (CPM)	RM1
Rad monitor steam line (CPM)	RM2
Rad rel rate condenser off-gas (GBq/s)	STTB
Rad rel rate reactor building(GBq/s)	STRB
Rad rel rate steam generator valves (GBq/s)	STSG
Ratio departure from nuclear boiling	DNBR
Reactivity fuel (doppler) (%dk/k)	RHFL
Reactivity mod temperature (%dk/k)	RHMT
Reactivity rod (%dk/k)	RHRD
Reactivity soluble boron (%dk/k)	RHBR
Reactivity total (%dk/k)	RH
Temp average fuel ($^{\circ }\text {C}$)	TF
Temp hot leg ($^{\circ }\text {C}$)	THA and THB
Temp loop subcooling margin ($^{\circ }\text {C}$)	SCMA and SCMB
Temp of debris in cavity ($^{\circ }\text {C}$)	TDBR
Temp of molten concrete ($^{\circ }\text {C}$)	TCRT
Temp of debris in lower plenum ($^{\circ }\text {C}$)	TSLP
Temp peak clad ($^{\circ }\text {C}$)	TPCT
Temp peak fuel ($^{\circ }\text {C}$)	TFPK
Temp Przr saturation ($^{\circ }\text {C}$)	TSAT
Temp reactor building ($^{\circ }\text {C}$)	TRB
Flow steam generator tube leak (t/hr)	WTRA and WTRB
Flow total break entering reactor building(t/hr)	WBK
Flow total emergency core cooling system (t/hr)	WECS
Fraction Zr oxidation	FRZR
Integrated break energy (MJ)	EBK
Temp submerged fuel average ($^{\circ }\text {C}$)	TFSB
Temperature cold leg ($^{\circ }\text {C}$)	TCA and TCB
Temperature reactor coolant system average ($^{\circ }\text {C}$)	TAVG
Volume reactor coolant system liquid ($\hbox {m}^3$)	VOL
Void of reactor coolant system (%)	VOID

## References

[1] Wang, Z., Wei, H., Tian, R. & Tan, S. A review of data-driven fault diagnosis method for nuclear power plant. *Progress Nucl. Energy* **186**, 105785. 10.1016/j.pnucene.2025.105785 (2025).

[2] Mendoza, M. & Tsvetkov, P. V. An intelligent fault detection and diagnosis monitoring system for reactor operational resilience: Power transient identification. *Progress Nucl. Energy* **156**, 104529. 10.1016/j.pnucene.2022.104529 (2023).

[3] Song, H., Liu, X. & Song, M. Comparative study of data-driven and model-driven approaches in prediction of nuclear power plants operating parameters. *Appl. Energy* **341**, 121077. 10.1016/j.apenergy.2023.121077 (2023).

[4] Hu, G., Zhou, T. & Liu, Q. Data-driven machine learning for fault detection and diagnosis in nuclear power plants: a review. *Front. Energy Res.* **9**, 663296. 10.3389/fenrg.2021.663296 (2021).

[5] Chae, Y. H., Koo, S. R., Choi, J., & Kim, J. Enhanced learning for nuclear power plant condition diagnoses using information metric based on Bayesian neural networks and UMAP. *Nucl. Eng. Technol.* **2025**, 103886. 10.1016/j.net.2025.103886 (2025).

[6] Choi, J., Koo, S. R. & Lee, S. J. Sensor fault detection and signal restoration using scenario-guided supervised autoencoders for nuclear power plant accidents. *Progress Nucl. Energy* **196**, 106374. 10.1016/j.pnucene.2026.106374 (2026).

[7] Stefenon, S. F., Seman, L. O. & Yow, K.-C. Graph attention network with mutual k-nearest neighbor strategy for predictive maintenance in nuclear power plants. *Technologies* **14**(1), 26. 10.3390/technologies14010026 (2026).

[8] Yin, W. et al. Early fault detection method for nuclear power plants based on sparse denoising autoencoder and kernel principal component analysis. *Ann. Nucl. Energy* **220**, 111460. 10.1016/j.anucene.2025.111460 (2025).

[9] Zhong, R. et al. Uncertainty-aware nuclear power turbine vibration fault diagnosis method integrating machine learning and heuristic algorithm. *IET Collab. Intell. Manufact.* **6**(3), 12108. 10.1049/cim2.12108 (2024).

[10] Ramezani, I., Vosoughi, N. & Ghofrani, M. B. Application of deep learning techniques for nuclear power plant transient identification. *Ann. Nucl. Energy* **194**, 110113. 10.1016/j.anucene.2023.110113 (2023).

[11] Li, J., Lin, M., Li, Y. & Wang, X. Transfer learning with limited labeled data for fault diagnosis in nuclear power plants. *Nucl. Eng. Design* **390**, 111690. 10.1016/j.nucengdes.2022.111690 (2022).

[12] Ayodeji, A., Amidu, M. A., Olatubosun, S. A., Addad, Y. & Ahmed, H. Deep learning for safety assessment of nuclear power reactors: reliability, explainability, and research opportunities. *Progress Nucl. Energy* **151**, 104339. 10.1016/j.pnucene.2022.104339 (2022).

[13] Wang, Y., Liu, J. & Qian, G. Hierarchical fft-lstm-gcn based model for nuclear power plant fault diagnosis considering spatio-temporal features fusion. *Progress Nucl. Energy* **171**, 105178. 10.1016/j.pnucene.2024.105178 (2024).

[14] Zhou, S., Lin, M., He, J., Wu, Y., & Wang, X. Unsupervised clustering research of nuclear power plants under unlabeled unknown fault diagnosis scenario. *Energy* **2025**, 136357. 10.1016/j.energy.2025.136357 (2025).

[15] Zhang, J., Xia, H., Zhu, Y. & Fu, Y. Research on sensor fault tolerance technology in nuclear power plant control system. *Ann. Nucl. Energy* **207**, 110714. 10.1016/j.anucene.2024.110714 (2024).

[16] Bae, J., Kim, K. S., & Lee, S. J. Two-stage deep learning model for nuclear power plant parameter trend prediction with epistemic uncertainty quantification. *Appl. Soft Comput.* **2025**, 114233. 10.1016/j.asoc.2025.114233 (2025).

[17] Lin, T.-H., Wang, T.-C. & Wu, S.-C. Deep learning schemes for event identification and signal reconstruction in nuclear power plants with sensor faults. *Ann. Nucl. Energy* **154**, 108113. 10.1016/j.anucene.2020.108113 (2021).

[18] Wang, X., Wang, H. & Peng, M. Interpretability study of a typical fault diagnosis model for nuclear power plant primary circuit based on a graph neural network. *Reliab. Eng. Syst. Saf.* **261**, 111151. 10.1016/j.ress.2025.111151 (2025).

[19] Qi, B., Liang, J. & Tong, J. Fault diagnosis techniques for nuclear power plants: a review from the artificial intelligence perspective. *Energies* **16**(4), 1850. 10.3390/en16041850 (2023).

[20] Qi, B., Zhang, L., Liang, J. & Tong, J. Combinatorial techniques for fault diagnosis in nuclear power plants based on bayesian neural network and simplified bayesian network-artificial neural network. *Front. Energy Res.* **10**, 920194. 10.3389/fenrg.2022.920194 (2022).

[21] Zhang, C., Chen, P., Jiang, F., Xie, J. & Yu, T. Fault diagnosis of nuclear power plant based on sparrow search algorithm optimized cnn-lstm neural network. *Energies* **16**(6), 2934 (2023).

[22] Yao, Y., Ge, D., Yu, J. & Xie, M. Model-based deep transfer learning method to fault detection and diagnosis in nuclear power plants. *Front. Energy Res.* **10**, 823395. 10.3389/fenrg.2022.823395 (2022).

[23] Dave, A. J., Nguyen, T. N., & Vilim, R. B. Integrating LLMs for explainable fault diagnosis in complex systems. arXiv preprint arXiv:2402.06695 1–4 (2024).

[24] Lin, M. et al. Generalization analysis and improvement of cnn-based nuclear power plant fault diagnosis model under varying power levels. *Energy* **282**, 128905. 10.1016/j.energy.2023.128905 (2023).

[25] Li, X., Huang, T., Cheng, K., Qiu, Z. & Sichao, T. Research on anomaly detection method of nuclear power plant operation state based on unsupervised deep generative model. *Ann. Nucl. Energy* **167**, 108785. 10.1016/j.anucene.2021.108785 (2022).

[26] Gao, J., Liu, Y., Zhao, Q., Ai, X. & Shan, L. Research on sensor anomaly detection and fault location in nuclear power plants based on gan-iaakr model. *Ann. Nucl. Energy* **219**, 111462. 10.1016/j.anucene.2025.111462 (2025).

[27] Wang, Z., Xia, H., Zhang, J., Yang, B. & Yin, W. Imbalanced sample fault diagnosis method for rotating machinery in nuclear power plants based on deep convolutional conditional generative adversarial network. *Nucl. Eng. Technol.* **55**(6), 2096–2106. 10.1016/j.net.2023.02.036 (2023).

[28] Li, Q. et al. Fault diagnosis of nuclear power plant sliding bearing-rotor systems using deep convolutional generative adversarial networks. *Nucl. Eng. Technol.* **56**(8), 2958–2973. 10.1016/j.net.2024.02.056 (2024).

[29] Guo, J., Wang, Y., Sun, X., Liu, S. & Du, B. Imbalanced data fault diagnosis method for nuclear power plants based on convolutional variational autoencoding wasserstein generative adversarial network and random forest. *Nucl. Eng. Technol.* **56**(12), 5055–5067. 10.1016/j.net.2024.07.015 (2024).

[30] Zheng, Q. et al. The real-time detection of defects in nuclear power pipeline thermal insulation glass fiber by deep-learning. *Energy* **313**, 133774. 10.1016/j.energy.2024.133774 (2024).

[31] Li, J., Lin, M., Li, Y. & Wang, X. Transfer learning network for nuclear power plant fault diagnosis with unlabeled data under varying operating conditions. *Energy* **254**, 124358. 10.1016/j.energy.2022.124358 (2022).

[32] Fang, J., Li, S., Wu, Y., He, M. & Xu, F. Research on an intelligent fault diagnosis method for nuclear power plants based on etcn-ssa combined algorithm. *Ann. Nucl. Energy* **213**, 111138. 10.1016/j.anucene.2024.111138 (2025).

[33] Li, X. et al. Fault diagnosis to nuclear power plant system based on time-series convolution neural network. *Wirel. Commun. Mobile Comput.* **2022**(1), 3323239. 10.1155/2022/3323239 (2022).

[34] Wang, H. et al. Advanced fault diagnosis method for nuclear power plant based on convolutional gated recurrent network and enhanced particle swarm optimization. *Ann. Nucl. Energy* **151**, 107934. 10.1016/j.anucene.2020.107934 (2021).

[35] Ayodeji, A., Liu, Y.-K. & Xia, H. Knowledge base operator support system for nuclear power plant fault diagnosis. *Progress Nucl. Energy* **105**, 42–50. 10.1016/j.pnucene.2017.12.013 (2018).

[36] Wang, H. et al. Nuclear power systems unsupervised anomaly localization considering spatiotemporal information and influence mechanism between devices. *Energy* **325**, 136204. 10.1016/j.energy.2025.136204 (2025).

[37] Liu, S. et al. Graph attention network-based model for multiple fault detection and identification of sensors in nuclear power plant. *Nucl. Eng. Design* **419**, 112949. 10.1016/j.nucengdes.2024.112949 (2024).

[38] Zhang, L. et al. System-level anomaly detection for nuclear power plants using variational graph auto-encoders. In *2021 IEEE International Conference on Sensing, Diagnostics, Prognostics, and Control (SDPC), vol. 1, pp. 180–185. IEEE, Weihai, China* (2021). 10.1109/SDPC52933.2021.9563515.

[39] Qu, Y., Xue, X. & Shi, Y. Graph classification based fault detection in nuclear power plants with graph formulation. *Procedia Comput. Sci.* **221**, 657–663. 10.1016/j.procs.2023.08.035 (2023).

[40] Guohua, W., Diping, Y., Jiyao, Y., Yiqing, X. & Dongxu, J. A framework for monitoring and fault diagnosis in nuclear power plants based on signed directed graph methods. *Front. Energy Res.* **9**, 641545. 10.3389/fenrg.2021.641545 (2021).

[41] Zhang, S. et al. Integration of multi-relational graph oriented fault diagnosis method for nuclear power circulating water pumps. *Measurement* **242**, 115811. 10.1016/j.measurement.2024.115811 (2025).

[42] International Atomic Energy Agency, I.A.E.A.: PCTRAN generic pressurized water reactor simulator exercise handbook. https://www-pub.iaea.org/MTCD/Publications/PDF/TCS-68web.pdf (2019).

[43] Mandal, S., Santhi, B., Sridhar, S., Vinolia, K. & Swaminathan, P. Sensor fault detection in nuclear power plant using statistical methods. *Nucl. Eng. Design* **324**, 103–110. 10.1016/j.nucengdes.2017.08.028 (2017).

[44] Zhu, S. et al. A robust strategy for sensor fault detection in nuclear power plants based on principal component analysis. *Ann. Nucl. Energy* **164**, 108621. 10.1016/j.anucene.2021.108621 (2021).

[45] Akter, S. et al. Comparative analysis of thermal hydraulic parameters of AP-1000 and VVER-1200 nuclear reactor for turbine trip concurrent with anticipated transient without SCRAM (ATWS). In *International Conference on Automation, Control and Mechatronics for Industry 4.0 (ACMI), pp. 1–6. IEEE, Rajshahi, Bangladesh* (2021). 10.1109/ACMI53878.2021.9528212.

[46] Wang, L., Chen, C., Chen, L., Li, Z. & Zeng, W. A coordinated control methodology for small pressurized water reactor with steam dump control system. *Energy* **282**, 129008. 10.1016/j.energy.2023.129008 (2023).

[47] Ren, C. et al. A cnn-lstm-based model to fault diagnosis for cpr1000. *Nucl. Technol.* **209**(9), 1365–1372. 10.1080/00295450.2023.2199098 (2023).

[48] Stefenon, S. F. et al. Group method of data handling using Christiano-Fitzgerald random walk filter for insulator fault prediction. *Sensors* **23**(13), 6118. 10.3390/s23136118 (2023). PMC10346365 37447968

[49] Zhao, X., Wang, X. & Golay, M. W. Bayesian network-based fault diagnostic system for nuclear power plant assets. *Nucl. Technol.* **209**(3), 401–418. 10.1080/00295450.2022.2142445 (2023).

[50] Choi, J. & Lee, S. J. RNN-based integrated system for real-time sensor fault detection and fault-informed accident diagnosis in nuclear power plant accidents. *Nucl. Eng. Technol.* **55**(3), 814–826. 10.1016/j.net.2022.10.035 (2023).

[51] Seman, L. O. et al. Simultaneously anomaly detection and forecasting for predictive maintenance using a zero-cost differentiable architecture search-based network. *Comput. Ind. Eng.* **2025**, 111412. 10.1016/j.cie.2025.111412 (2025).

[52] Starke, L. et al. Interference recommendation for the pump sizing process in progressive cavity pumps using graph neural networks. *Sci. Rep.* **13**(1), 16884. 10.1038/s41598-023-43972-4 (2023). PMC10558576 37803055

[53] Zhang, Z., Yuan, G., Qin, Z., & Luo, Q. An improvement by introducing lbfgs idea into the adam optimizer for machine learning. *Expert Syst. Appl.* **2025**, 129002. 10.1016/j.eswa.2025.129002 (2025).

[54] Coelho, R. C., Alves, A. F. C., Pires, T. N. & Pires, F. A. A composite Bayesian optimisation framework for material and structural design. *Comput. Methods Appl. Mech. Eng.* **434**, 117516. 10.1016/j.cma.2024.117516 (2025).

[55] Cowen-Rivers, A. et al. Hebo: pushing the limits of sample-efficient hyperparameter optimisation. *J. Artif. Intell. Res.* **74**, 1269–1349 (2022).

[56] Liu, J., Hou, B., Lu, M. & Wang, D. Box-cox transformation based state-space modeling as a unified prognostic framework for degradation linearization and rul prediction enhancement. *Reliab. Eng. Syst. Saf.* **244**, 109952. 10.1016/j.ress.2024.109952 (2024).

[57] Yang, L., Nguyen-Thoi, T. & Tran, T.-T. Predicting the friction angle of clays using a multi-layer perceptron neural network enhanced by yeo-johnson transformation and coral reefs optimization. *J. Rock Mech. Geotech. Eng.* **16**(10), 3982–4003. 10.1016/j.jrmge.2024.02.025 (2024).

[58] Stefenon, S. F., Cristoforetti, M. & Cimatti, A. Automatic digitalization of railway interlocking systems engineering drawings based on hybrid machine learning methods. *Expert Syst. Appl.* **281**, 127532. 10.1016/j.eswa.2025.127532 (2025).

[59] Juarsa, M. et al. An alternative cooling solution during loss of coolant accident with emergency core cooling system failure in nuscale nuclear reactor. *Progress Nucl. Energy* **169**, 105063. 10.1016/j.pnucene.2024.105063 (2024).

[60] Farber, J. A. & Cole, D. G. Detecting loss-of-coolant accidents without accident-specific data. *Progress Nucl. Energy* **128**, 103469. 10.1016/j.pnucene.2020.103469 (2020).

[61] Zhang, J. et al. Remaining useful life prediction based on interpretable serialized variational autoencoder: a drift-diffusion stochastic equation perspective. *IEEE Trans. Ind. Inf.* **2026**, 1–12. 10.1109/TII.2026.3657827 (2026).

[62] Zhang, J., Wang, C., Qian, Q., & Yin, S. Source-free domain adaptation for cross-domain remaining useful life prediction: a distributed federated learning perspective. *Reliab. Eng. Syst. Saf.* **2026**, 112271. 10.1016/j.ress.2026.112271 (2026).

[63] Tian, J. et al. Federated transfer ensemble learning with mixup-augmented domain adversarial adaptation network for cross-domain fault diagnosis. *Control Eng. Pract.* **173**, 106999. 10.1016/j.conengprac.2026.106999 (2026).

[64] Qian, Q., Zhou, J., Hou, B., Wang, J., Sheng, H., & Zhang, J. Dimension-mismatched adversarial network: a new feature distribution adaptation method for rolling bearing rul prediction. *Reliab. Eng. Syst. Saf.* **2026**, 112269. 10.1016/j.ress.2026.112269 (2026).

[65] Zhang, J. et al. Remaining useful life prediction based on self-attention mechanism-sequential variational autoencoder: From a semi-supervised perspective. *Adv. Eng. Inf.* **71**, 104242. 10.1016/j.aei.2025.104242 (2026).

